# CoSP: Reconfigurable Metamaterial Inverse Design via Contrastive Pretrained Large Language Model

**DOI:** 10.1002/advs.76917

**Published:** 2026-08-11

**Authors:** Shujie Yang, Yuqi Zhang, Xuzhe Zhao, Yansong Tang, Kaichen Dong

**Affiliations:** ^1^ Institute of Data and Information, Tsinghua Shenzhen International Graduate School Tsinghua University Shenzhen Guangdong China; ^2^ Center of Double Helix, Tsinghua Shenzhen International Graduate School Tsinghua University Shenzhen Guangdong China; ^3^ Intelligent Passive Thermal Control Center Research Institute of Tsinghua University in Shenzhen Shenzhen Guangdong China

**Keywords:** contrastive multi‐state pretrain, inverse design, large language models, reconfigurable metamaterials

## Abstract

Metamaterials for light manipulation at subwavelength scales face significant design challenges due to their complex and sophisticated structures, leading to the emergence of deep learning as a powerful tool to streamline their design process. However, existing deep learning‐based inverse design methods fall short in the design of reconfigurable metamaterials (RMMs), whose optical characteristics switch between different states upon external stimuli. To address this challenge, CoSP, an intelligent inverse design method for RMMs based on a contrastive pretrained large language model (LLM), is proposed. By performing contrastive pretraining on multi‐state spectra, a well‐trained spectrum encoder is obtained and coupled to a GPT‐style decoder trained end‐to‐end from scratch. Equipped with the preservation of linguistic capabilities, CoSP is capable of describing material structures with target optical properties in natural language. Numerical experiments demonstrate that CoSP can design RMM structures for multi‐state, multi‐band optical responses, showing great potential in versatile applications such as thermal management, optical computation, and telecommunications.

## Graphical abstract

In this work, CoSP (contrastive multi‐state pretrain), an intelligent inverse design method for reconfigurable metamaterials based on a contrastive pretrained large language model, is proposed. Numerical experiments demonstrate that CoSP can design reconfigurable metamaterial structures for multi‐state, multi‐band optical responses, showing great potential in versatile applications such as thermal management, optical computation, and telecommunications.

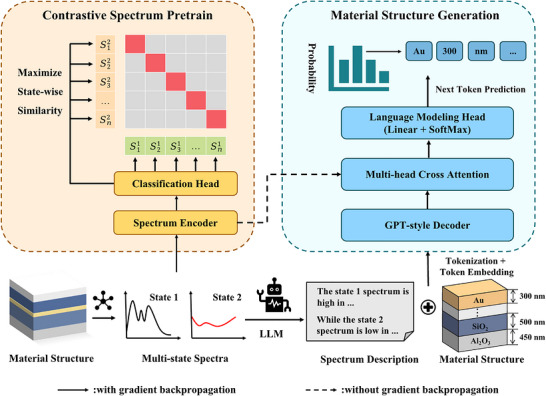

## 1 Introduction

Metamaterials [1, 2, 3], which are composed of subwavelength structures, have introduced a novel paradigm for designing artificial materials with extraordinary properties that are scarce or even impossible in natural materials. By precisely engineering their subwavelength structures [4], metamaterials exhibit almost arbitrarily desired properties for industrial applications, including information sciences [5], optoelectronics [6], biomedical sciences [7], and energy‐saving technologies [8]. However, the intricate design of metamaterials, which involves carefully adjusting sizes and arrangements of every individual subwavelength structure through trial and error, is extremely time‐consuming and difficult even for experienced researchers. Moreover, traditional optimization methods for metamaterials rely heavily on exhaustive parameter sweeps and heuristic searches with Finite Difference Time Domain (FDTD) and Finite Element Method (FEM), making them inefficient and computationally demanding in complex, high‐dimensional design spaces. Furthermore, such experience‐driven approaches also lack transferability among development projects and disciplines.

**FIGURE 1:**
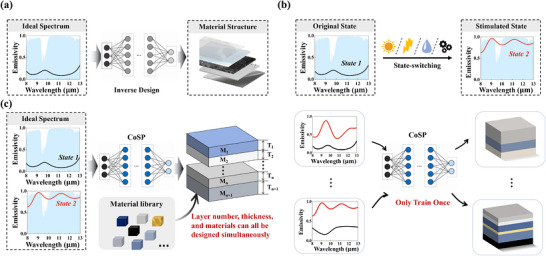
Schematic illustration of the problem setting and the advantages of CoSP. (a) General inverse design methods aim to generate metamaterial structures for one desired optical response, which are typically limited to single‐state targets, fixed material sets, or predefined layer configurations. (b) RMMs exhibit distinct spectral responses under external stimuli. (c) CoSP enables inverse design for multi‐state spectral responses in RMMs with substantially greater flexibility: the layer number, thickness, and material can all be designed simultaneously, and a single trained model can generalize to multiple spectral‐response objectives without task‐specific retraining.

Thanks to the fast development of artificial intelligence (AI), inverse design methods have emerged as a novel solution to metamaterials design and optimization [9]. Rooted in models that can learn the intricate relationships between metamaterial structures and their optical responses, inverse design focuses on the data‐driven discovery of optimal material compositions and structural configurations for desired optical properties. So far, various AI‐empowered approaches, including Bayesian optimization [10], deep learning [9], and reinforcement learning [11], have already accelerated the discovery of novel metamaterials by intelligently navigating vast design spaces, as illustrated in Figure 1A.

Reconfigurable metamaterials [12, 13] (RMMs) exhibit distinct optical properties at different states driven by external stimuli such as electricity, heat, humidity, and mechanical forces [11, 14], as shown in Figure 1B. This dynamic tunability makes RMMs particularly attractive for intelligent applications that require distinct device performance under varying conditions. However, compared with conventional metamaterials with fixed optical responses, RMMs impose a more demanding inverse design problem: a single structure must achieve multiple target responses across different states. This requirement calls for both sufficient design flexibility and the ability to adapt across variable design objectives.

Despite substantial progress in AI‐based inverse design for static metamaterials, existing methods remain inadequate for RMMs for two main reasons: (1) they provide limited design freedom: the models are restricted to fixed material sets and predefined structural configurations [15, 16]; (2) they show poor transferability: when the design objective or material composition changes, the models require extensive retraining or task‐specific reconfiguration [10, 17, 18]. Consequently, none of the existing inverse design methods [10, 11, 14, 15, 16, 17, 18] demonstrates effective inverse design of RMMs. Figure 1 illustrates the above concepts about RMMs and the shortcomings of existing inverse design methods. This problem severely limits the development of RMMs and other intelligent reconfigurable devices.

In this paper, CoSP (contrastive multi‐state pretrain), an inverse design method for reconfigurable devices via a contrastive pretrained large language model (LLM), is proposed. Unlike prior methods, CoSP employs contrastive learning to unify multi‐state spectral representations, explicitly capturing the intrinsic physical correlations between different states of the same device including RMMs. These robust embeddings are then fed into a GPT‐style decoder trained end‐to‐end from scratch, which synergizes its generative linguistic capabilities with physical understanding to simultaneously output human‐readable spectrum descriptions and precise material structures. This architecture enables CoSP to generalize across multi‐state, multi‐band targets without task‐specific retraining. Extensive experiments validate that CoSP can successfully design RMMs even from hypothetical spectra, marking a transformative step toward data‐driven fast development of intelligent reconfigurable devices.

## 2 Related Works

### 2.1 Reconfigurable Metamaterials

The emergence of the metamaterials concept and the development of nanofabrication technologies have enabled the creation of artificial materials with desired physical properties. However, conventional metamaterials are designed with fixed physical properties, which fail to offer adjustable device functionalities for emerging intelligent industrial applications, such as smart windows with adjustable visible transparency [19], temperature‐adaptive emissive coatings for all‐season temperature regulation [20], reconfigurable photonic integrated circuits for optical computation [21], and solid‐state LiDAR [22]. To meet the above requirements, the concept of RMMs was proposed [6].

The specific implementations of RMMs are diverse: In electrically driven RMMs, reversible metal deposition methods [23] are widely used to achieve rapid optical property switching of the metamaterial. Mechanically driven RMMs mostly rely on temperature‐responsive materials or electrostatic force to reconfigure mechanical structures for mode‐switching [24]. Humidity‐driven RMMs achieve switching by designing hydrophobic and hydrophilic properties [25]. Thermally driven RMMs depend on thermochromic hydrogels [26] or phase‐change materials [27]. Without loss of generality, this paper takes thermally driven RMMs integrated with temperature‐responsive phase‐change materials as an example: With the ability to automatically adjust the broadband thermal emissivity according to environmental temperature change, those RMMs have been widely applied to various industrial fields, including smart windows [28], adaptive radiative cooling [20], and infrared camouflage [29], with a broad market potential.

### 2.2 Intelligent Inverse Design of Metamaterials

Different AI‐empowered approaches have been used to design conventional metamaterials with fixed functionalities: Sakurai et al. [10] used a Bayesian Optimization (BO) method to optimize layer thicknesses for ultranarrow‐band thermal radiators, which offers great material and structural flexibility but lacks task adaptability and generalization. Unni et al. [15] reported a Mixture Density Networks (MDNs) model to address non‐uniqueness through probabilistic modeling, but it cannot dynamically adjust the number of layers or generalize across different tasks. Existing inverse‐design models based on Reinforcement Learning, such as Yu et al. [17] and Wang et al. [11], improve material and structural flexibility, but are incapable of handling multi‐state responses and require retraining for new tasks. Material informatics methods, like Xi et al. [18], solve multi‐objective tasks like visible‐infrared camouflage but are limited by fixed layer configurations and poor generalization. Although Transformer‐based models improve adaptability—for example, Chen et al. [16] for broadband absorber design—they lack material and structural flexibility. OptoGPT by Ma et al. [14] advances task transferability and generalization through flexible material and layer design. However, despite adopting large language model (LLM) structures, OptoGPT focuses solely on structure prediction and lacks the ability to generate human‐readable explanations of spectral data. Taken together, these studies have substantially advanced the inverse design of metamaterials, but the joint realization of paired state‐dependent responses in one reconfigurable metamaterial, flexible material–layer generation, and human‐readable spectral output remains unexplored.

In contrast, the proposed CoSP framework not only enables the inverse design of RMMs, but also handles multi‐state responses in a human‐readable manner. Table 1 compares the capabilities of CoSP with the aforementioned intelligent inverse design methods, whose associated method categories are provided in parentheses. Those methods were developed under different design objectives and structural representations: probabilistic models address the non‐uniqueness of inverse mappings; optimization and reinforcement‐learning methods provide flexible search strategies within their respective parameter and objective settings; Transformer‐based generative models enable sequence‐based structure generation. Their reported capabilities therefore reflect the specific conditioning inputs, structural parameterizations, and output formats considered in the corresponding studies. However, none of them was formulated for the inverse design of RMMs, where paired state‐dependent target spectra must be realized by one metamaterial structure. As shown in Table 1, CoSP integrates all capabilities within a unified inverse‐design framework. A more detailed dimension‐by‐dimension elaboration of these advantages is provided in Section S1.

**TABLE 1:** Comparison between CoSP and existing inverse design methods for metamaterials.

Model	Multi‐band	Material	Layer	Transferability	Generality	Multi‐state	Linguistic
Unni et al. [15] (MDN)							
Chen et al. [16] (MST)	✓						
Sakurai et al. [10] (BO)		✓	✓				
Xi et al. [18] (MI‐BO)	✓		✓				
Yu et al. [17] (DQN)	✓	✓		✓			
Wang et al. [11] (DRL)	✓	✓	✓	✓			
Ma et al. [14] (OptoGPT)	✓	✓	✓	✓	✓		
**This work (CoSP)**	✓	✓	✓	✓	✓	✓	✓

**Multi‐band** means the model can design metamaterials that function across multiple spectral regions. **Material** means the model can freely select and optimize various materials without being limited to predefined sets. **Layer** refers to the dynamical adjustment of the number of layers in the structure for greater design flexibility. **Transferability** denotes the ability to adapt to new tasks by retraining with updated objective functions. **Generality** indicates the model can design structures for arbitrary objectives without retraining. **Multi‐state** means the model can design RMMs with distinct optical properties in different states. **Linguistic** is the ability to generate human‐readable descriptions using natural languages, improving interpretability and usability.

### 2.3 Contrastive Representation Learning

Contrastive learning [30, 31, 32] is a self‐supervised representation learning method that aims to maximize the similarity between positive sample pairs while minimizing the similarity between negative sample pairs, thereby facilitating robust feature extraction without extensive labeled data. Contrastive Predictive Coding (CPC) [33] introduces the concept of predicting future data points in latent space, thus learning effective representations by capturing temporal dependencies within sequential data. Building on this, SimCLR [34] enhances contrastive learning by utilizing a simplified framework that applies extensive data augmentation and a large batch size to effectively discriminate between similar and dissimilar pairs. Momentum Contrast (MoCo) [35] incorporates a dynamic memory bank, enabling the model to maintain a large and consistent set of negative samples, thus enhancing training efficiency and representation quality. CLIP [36] pushes the boundaries of contrastive learning by leveraging cross‐modal training on extensive image‐text datasets, resulting in representations that align across modalities and achieve remarkable performance on a wide range of downstream tasks. The above characteristics of contrastive learning demonstrate its great potential for learning unified representations across the multiple states of RMMs and motivate its adoption in the CoSP framework.

### 2.4 Large Language Model for Natural Sciences

Large language models (LLMs) have been increasingly utilized in various natural science domains [37, 38, 39, 40, 41, 42], such as bioinformatics [43], materials science [14, 44], and chemistry [45, 46, 47], demonstrating powerful capabilities in AI‐for‐Science tasks. For example, Prot2Text [48] employs the multi‐modal information of proteins to fine‐tune LLMs, enabling the generation of detailed protein descriptions; OptoGPT [14] leverages LLMs to design thin‐film materials; Coscientist [47] utilizes LLMs to automate chemical experiments, where humans can simply issue instructions in natural language to an LLM‐controlled robotic system for searching information, conducting experiments, recording data, and analyzing results. These applications highlight the robust capabilities and great potential of LLMs in the natural sciences. Therefore, the integration of LLMs into scientific workflows promises to accelerate discovery processes, enhance the precision of experimental designs, and potentially uncover novel scientific insights that were previously unattainable through traditional methods. Recent studies have begun to explore their potential in metamaterial design. Kim et al. demonstrated conversational optimization of multilayer films and fine‐tuned LLM‐based generation of metasurface structures [49], in which the two physical systems were addressed through separate workflows. Lu et al. investigated the ability of ChatGPT to learn geometry–spectrum relationships in metamaterials and examined both forward and inverse mappings within a predefined structural representation [50]. Zhang et al. further fine‐tuned an LLM to translate target transmission spectra into free‐form 2D metasurface geometries on a fixed material platform [51]. Together, these studies establish the potential of LLMs for optical‐response modeling and photonic structure generation. Unlike these single‐response formulations, CoSP focuses on the generation of reconfigurable metamaterial on paired state‐dependent spectra and jointly produces the corresponding spectral description and material–structure sequence. Motivated by these advances, a GPT‐style decoder is adopted as the generative backbone of the inverse design framework for three reasons: (1) LLMs possess strong natural‐language generation capabilities, enabling the model to produce human‐readable spectrum descriptions alongside material structures; (2) LLMs are capable of internalizing broad world knowledge spanning physics, materials science, and mathematics, potentially providing a rich prior for understanding the correspondence between optical spectra and metamaterial compositions; (3) the well‐established scaling laws of LLMs suggest that performance can be further improved simply by adopting larger models in the future, offering a clear path for continued advancement.

## 3 Methodology

**FIGURE 2:**
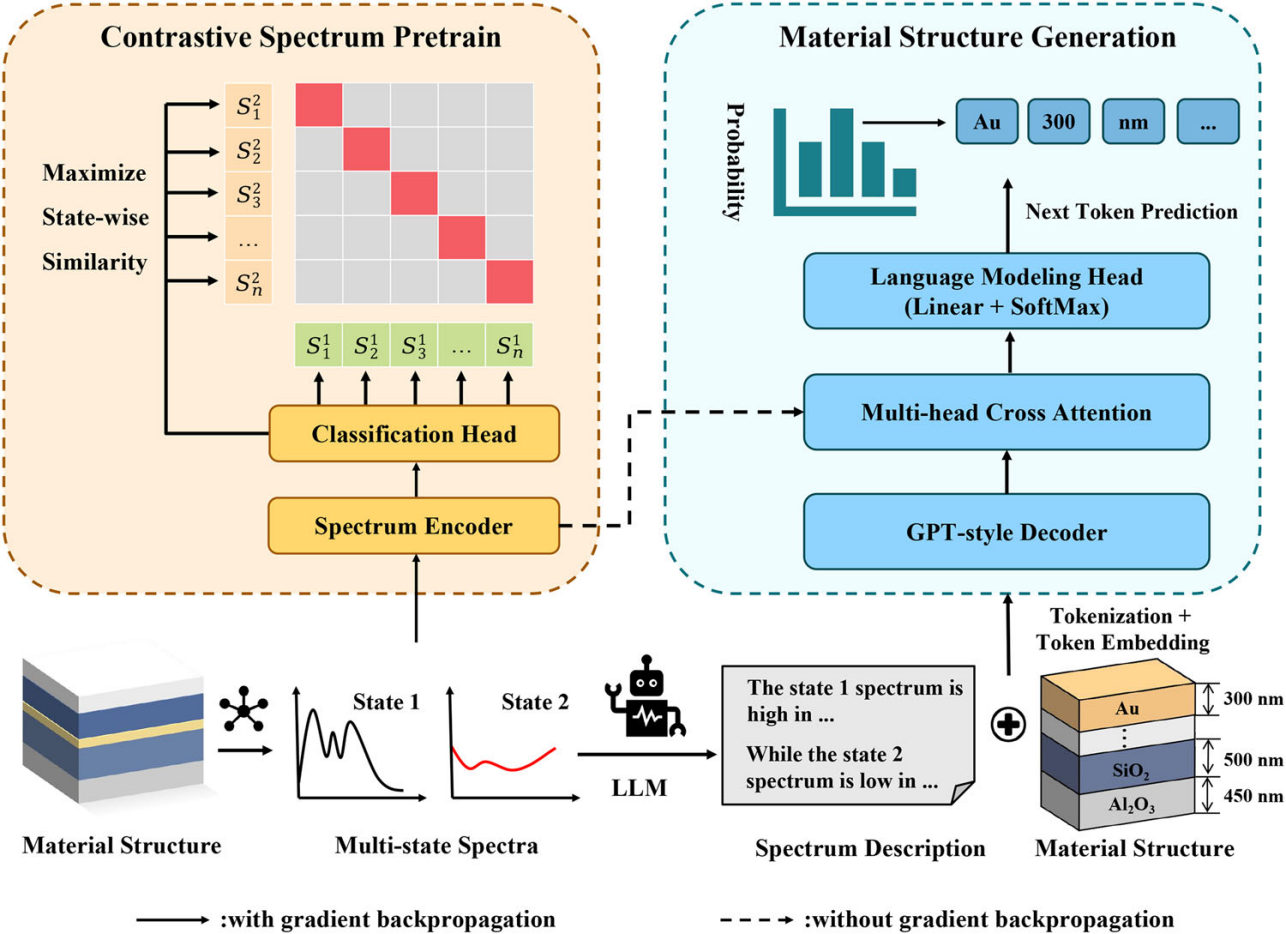
Architecture of the proposed CoSP framework for generating RMMs with multi‐state optical responses in free text sequence. The model is structured with an encoder‐decoder architecture, where the encoder utilizes contrastive learning for pretraining, integrating multi‐state spectral information by maximizing the cosine similarity of spectrum representations from different states of the same metamaterial. After contrastive pretraining, the spectrum encoder parameters are frozen. The framework employs a GPT‐style decoder to generate detailed and accurate spectrum descriptions and corresponding material structures from the representation of the target multi‐state spectra. By combining the capabilities of contrastive pretraining and LLMs, the model enables a comprehensive representation of multi‐state spectral characteristics, facilitating the inverse design of RMMs.

The switchable optical responses (such as optical absorptivity, reflectivity, and transmissivity) of RMMs impose a significant burden on their intelligent inverse design, as existing methods are unable to simultaneously address multiple target spectra of the same metamaterial. To tackle this challenge, the proposed CoSP consists of two stages (Figure 2): (1) contrastive multi‐state spectrum pretraining; (2) metamaterial structure generation with a pretrained spectrum encoder. For each target spectrum, the pretrained spectrum encoder obtains a representation, which then passes through cross‐attention into the LLM blocks. In this work, GPT‐2 was used for the generative model due to two reasons: (1) Its tokenizer, hidden states, and decoder blocks are fully accessible and can be modified directly, enabling cross‐attention to be integrated and optimized end‐to‐end in a reproducible implementation. (2) Its moderate model size provides an economical and computationally accessible backbone with effective performance. It is expected that CoSP integrated with recently released LLMs with openly accessible architecture will have even better performance, which is favorable for RMM inverse design tasks requiring higher performance and increased costs can be tolerated. After decoding, CoSP generates the description of the target spectra as well as the corresponding material compositions and structural configurations.

### 3.1 Contrastive Multi‐State Spectrum Pretraining

A Transformer encoder [52] was utilized as the backbone of the spectrum encoder, including wavelength embedding, positional encoding, multi‐head self‐attention, and feed‐forward networks. Notably, spectra are continuous across wavelength bands, in stark contrast to the discrete representation of text in lexical space. Token embedding may disrupt the spectrum continuity determined by physics, leading to unwanted segmented interpretation of spectral input as well as inverse design performance degradation. Therefore, in contrast to the text encoder used in CLIP [36], CoSP uses wavelength encoding instead of conventional token embedding.

Initially, the spectrum $S_{i}^{j}$ of different states j of the same metamaterial i is projected by a linear layer Proj. Then, the wavelength encoding WavEncoding is subsequently applied to the projected spectrum representation. In the absence of token embedding, the wavelength encoding is an absolute positional encoding applied in the sequence direction. Subsequently, the spectrum representation is processed through the N‐layer transformer encoder blocks H, yielding the output. The encoder blocks are followed by a fully connected layer ClsHead, the classification head, used solely during the contrastive pretraining stage. According to SimCLR [34], using a nonlinear fully connected layer (i.e., one that includes an activation function) leads to more effective representations than a linear fully connected layer. This process can be formally described by

(1) $$\begin{array}{cc} s_{i}^{j} & =Proj(S_{i}^{j})\\ s_{i}^{j} & =s_{i}^{j}+WavEncoding\left(s_{i}^{j}\right)\\ s_{i}^{j} & =ClsHead(H\left(s_{i}^{j}\right)). \end{array}$$

Then, this approach employs contrastive learning to maximize the similarity of spectrum representations $s_{i}^{j}$ of different states j of the same metamaterial i through the InfoNCE (Information Noise Contrastive Estimation) loss:

(2) $$\begin{array}{cc} L_{j}=-\frac{1}{n}\sum_{i=1}^{n}\log & \frac{\exp(\cos\left(s_{i}^{j},s_{i}^{j^{*}}\right)/\tau )}{\sum_{k=1}^{n}\exp\left(\cos\left(s_{i}^{j},s_{k}^{j^{*}}\right)/\tau \right)}\\ L= & \frac{1}{2}\left(L_{1}+L_{2}\right) \end{array}$$

where n is the batch size, $j^{*}$ denotes another state distinct from j, τ is a learnable temperature parameter to control the distribution of the similarity matrix. Thereby, a spectrum encoder is trained in a self‐supervised manner.

### 3.2 Spectrum Description

Due to the one‐to‐many mapping from optical spectra to metamaterial designs, different metamaterial designs may exhibit similar optical spectra. As a result, the training targets of inverse design are not limited to material sequences alone. Otherwise, the model is confined to the metamaterial design space within the training set, thus losing its generalization capability for new but similar spectra. Translating target spectra into human‐readable descriptions can significantly improve the generalization of the inverse design method, because all similar spectra can be recognized by the model to avoid the retraining process. Moreover, by observing how CoSP describes spectra in natural language, it is easy to audit its faithfulness to the input spectra. The effectiveness of the spectrum representations learned by the encoder can also be manually validated by researchers.

Hence, unlike existing inverse design methods that solely and directly generate metamaterial designs, CoSP is preceded by an LLM‐enabled spectrum description stage that depicts the target multi‐state spectra. It encompasses the local maxima and minima (i.e., peaks and troughs) of the spectra across different states and wavelength bands. The detailed generation process and data examples are provided in Section S3, and an ablation study confirming the benefit of incorporating spectrum description into training is reported in Section S2.

### 3.3 Inverse Design—Material Structure Generation

The Transformer decoder architecture is utilized to generate the structures in RMMs for targeted optical responses. The main components of the decoder—namely, the text embedding matrix, self‐attention mechanism, and language modeling head—are initialized with the configs of GPT‐2 [37]. As such, the ability of GPT‐2 is leveraged to understand the underlying textual semantics. The spectrum representation, obtained from an encoder pretrained through spectrum contrast, is fed into the multi‐head cross‐attention module within the Transformer decoder. The above process can be described by

(3) $$\begin{array}{cc} Z & =\text{Softmax}\left(\frac{QK^{⊤}}{\sqrt{d_{k}}}\right)V\\ Q & =W_{q}q,\;K=W_{k}s,\;V=W_{v}s \end{array}$$

where Q denotes the query matrix obtained by projecting the text embedding q with $W_{q}$. The key matrix K and value matrix V are computed by applying $W_{k}$ and $W_{v}$ to the spectral representation s, respectively. Through this multi‐head cross‐attention mechanism, the decoder conditions text generation on the encoded spectral representation, and enables the production of coherent and physically consistent descriptions of metamaterial structures for the target optical responses. In this way, CoSP captures the correspondence between optical spectra and metamaterial structures guided by the underlying physics.

During the training phase, Causal Language Modeling (CLM) is also employed to predict the next token in a sequence based on the preceding tokens, as the training objective to optimize the model as follows:

(4) $$L_{\text{CLM}}=-\sum_{i=1}^{n}\log P\left(x_{i}|x_{1},x_{2},\text{…},x_{i-1}\right)$$

where $L_{\text{CLM}}$ is the loss function for CLM. This training objective involves predicting each token $x_{i}$ in a sequence based on the preceding tokens $x_{1},x_{2},\text{…},x_{i-1}$, thereby ensuring that text generation occurs in a causal, forward manner. Precisely, this unidirectional autoregressive generation method aligns remarkably with the structures of RMMs. Detailed training and inference settings, including hyperparameters and hardware configurations, are provided in Section S4.

## 4 Results

### 4.1 Preliminary Setup

Without loss of generality, this work focuses on 1D RMMs integrated with the phase‐change material of vanadium dioxide (${\mathrm{VO}}_{2}$), in order to demonstrate the inverse design of multi‐state target spectra using CoSP: One‐dimensional metamaterials, featured by stacked thin‐film layers, are fundamental yet widely adopted metamaterial structures [10, 14, 20]; ${\mathrm{VO}}_{2}$ undergoes a fast, reversible, solid–solid phase transition when heated beyond 68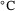, transforming from an insulating phase (**I‐phase**, corresponding to **State I** of RMMs) to a metallic phase (**M‐phase**, corresponding to **State M** of RMMs) [53]. Along with the above phase change, ${\mathrm{VO}}_{2}$ exhibits significant optical property contrast, endowing it with powerful optical sensing and modulation capabilities [20]. Thus, ${\mathrm{VO}}_{2}$‐based RMMs show great potential in versatile applications, including smart windows [28], temperature‐adaptive radiative coolers [20], reconfigurable phased arrays [54], and hyperspectral imaging [54].

In the experiments presented below, the absorptivity of stacked multilayer RMMs is taken as the optical response of interest. Since the sum of absorptivity, reflectivity, and transmissivity equals 1 [55], transmissivity is calculated as T(λ)=1−R(λ)−A(λ). Note that the optical refractive index of ${\mathrm{VO}}_{2}$ is taken from Ref. [56].

### 4.2 Main Results

#### 4.2.1 Comparison with Baselines

Table 2 summarizes the overall performance of CoSP against representative baselines spanning classical optimization, reinforcement learning, discriminative deep learning, and generative modeling. It is crucial to clarify that none of these baselines is originally designed to handle multi‐state RMM inverse design, where the objective is to simultaneously match a spectral pair corresponding to the two states of a single metamaterial structure. To ensure a fair and technically meaningful comparison, method‐specific adaptations are developed for every baseline, guided by the governing physics and the multi‐state objective. Concretely, each method is modified in a theory‐consistent manner (e.g., reformulating the optimization reward/loss to aggregate errors across both states and enforcing valid structural parameterizations), and these adaptations are tuned to ensure that each baseline can indeed generate candidate designs. As a result, all the values in Table 2 should be interpreted as the performance of solid, best‐effort adapted baselines, rather than off‐the‐shelf implementations. The full per‐method adaptation protocol is provided in Section S5.

**TABLE 2:** Performance comparison between CoSP and existing inverse design methods.

Method	MSE ↓	MAE ↓	RMSE ↓	*R* 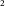 ↑	Corr. ↑	SID ↓
Classical Optimization Methods						
Genetic Algorithm	0.0716	0.1948	0.2466	−0.2994	0.5422	1.5251
Bayesian Optimization	0.1278	0.2540	0.3163	−1.2185	0.4286	2.8911
Reinforcement Learning Methods						
DQN	0.2537	0.4218	0.4839	−3.6053	0.3826	5.7245
PPO	0.2405	0.4092	0.4728	−3.4968	0.0347	4.5876
Discriminative Deep Learning Methods						
MLP	0.1225	0.2628	0.3291	−1.2174	0.3273	2.4536
CNN	0.1162	0.2522	0.3147	−1.0439	0.3636	2.3610
ResNet	0.1159	0.2523	0.3152	−1.0576	0.3680	2.3258
Transformer Encoder	0.1225	0.2653	0.3258	−1.1329	0.3801	2.4327
Generative Methods						
cGAN	0.0909	0.2217	0.2795	−0.6560	0.4510	1.9750
cVAE	0.0678	0.1876	0.2370	−0.1720	0.5320	1.3100
OptoGPT	0.2401	0.4016	0.4676	−3.3637	0.2189	5.1817
**CoSP (This work)**	**0.0342**	**0.1181**	**0.1585**	**0.4290**	**0.7216**	**0.3686**
**Improvement**	**+49.6%**	**+37.0%**	**+33.1%**	N/A	**+35.6%**	**+71.9%**

The best results are highlighted in **bold**. SID denotes Spectral Integral Difference. Improvement is calculated relative to the strongest baseline (cVAE). Note that the improvement for $R^{2}$ is marked as N/A because calculating a percentage increase from a negative baseline (cVAE: −0.17) to a positive value (CoSP: 0.43) is mathematically ill‐defined. Notably, CoSP is the only method to achieve a positive $R^{2}$, demonstrating predictive capability superior to a simple mean‐value predictor, whereas all baselines fail to capture the underlying data distribution.

Under this stringent evaluation protocol, CoSP achieves the best results across all criteria, establishing a clear margin over prior adapted inverse‐design strategies. Compared with the strongest adapted baseline, the conditional VAE (cVAE), CoSP reduces pointwise regression errors substantially, with MSE reduced from 0.0678 to 0.0342 (+49.6%), MAE from 0.1876 to 0.1181 (+37.0%), and RMSE from 0.2370 to 0.1585 (+33.1%). Beyond magnitude‐based metrics, CoSP also improves spectral fidelity in a stricter distributional sense: SID drops from 1.3100 to 0.3686 (+71.9%), indicating markedly better agreement in global spectral shape rather than merely local amplitude fitting. Consistently, CoSP attains a substantially higher correlation (0.7216 vs. 0.5320, +35.6%), and is the only method that yields a positive $R^{2}$ (0.4290), whereas all adapted baselines underperform a constant mean predictor on the test distribution with a negative $R^{2}$.

A cross‐family comparison further reveals why existing methods are inadequate for this multi‐state inverse‐design task, even after careful adaptation. Classical optimizers (GA/BO) are limited by search efficiency in a high‐dimensional, multi‐objective landscape and tend to produce designs with suboptimal distributional alignment (worse SID). Reinforcement learning methods (DQN/PPO) face pronounced instability and reward–objective mismatch when the reward must jointly capture two‐state spectral agreement, resulting in the largest errors and weak correlation. Discriminative predictors (MLP/CNN/ResNet/Transformer) achieve moderate pointwise errors but still yield negative $R^{2}$ and worse SID. This phenomenon is consistent with their one‐to‐many nature of inverse mapping, where deterministic regression collapses multimodal solutions and degrades spectral structure. Among generative baselines, cGAN [57] and cVAE [58] provide the strongest competitors: by explicitly modeling the conditional distribution of structures given a target spectrum, both methods cope better with the one‐to‐many nature of inverse design than deterministic regressors, with cVAE in particular reaching the lowest MSE/MAE/RMSE and SID among all baselines. Nevertheless, both still rely on a fixed‐length structural vector and a noise‐driven sampling procedure that lacks an explicit multi‐state representation, which limits their pointwise accuracy and leaves $R^{2}$ negative. In contrast, CoSP utilizes contrastive multi‐state spectrum pretraining to learn transferable spectral representations and couples them with structured generation, thereby achieving both lower numerical error and superior distributional consistency across the two states of RMMs.

#### 4.2.2 Inverse Design

**FIGURE 3:**
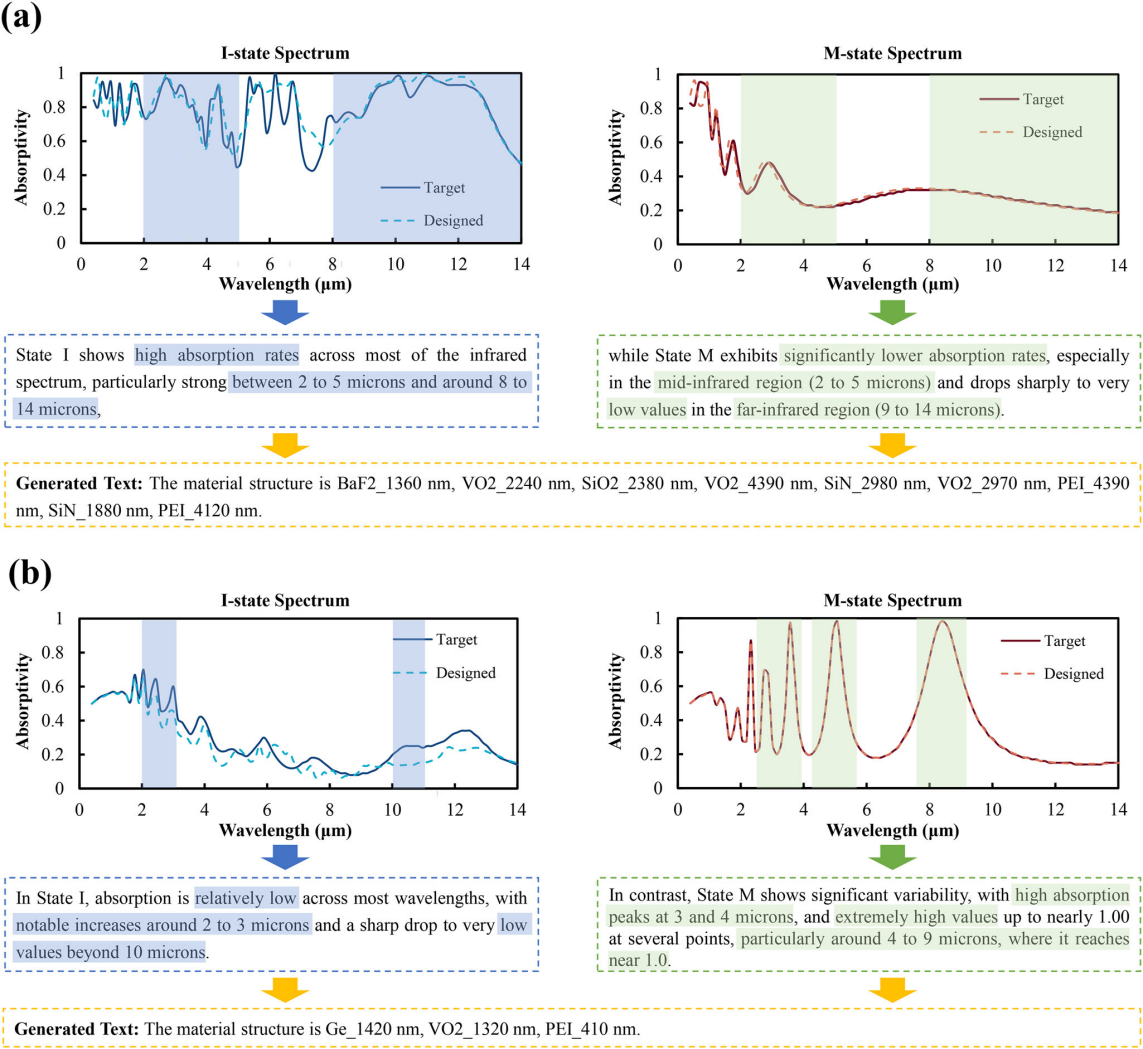
CoSP inverse design of RMMs. Target spectral pair represents the input two‐state spectra. Generated Text comprises the natural language results produced by CoSP, including the spectrum description and material structure sequence of the designed RMM. The designed spectra are calculated using TMM based on the RMM structure generated by CoSP. (a) and (b) are two representative samples.

In this section, two samples are selected from the test set to demonstrate the performance of CoSP. First, the two‐state absorptivity spectral pair is provided as the target. Subsequently, CoSP generates spectral descriptions and corresponding material structures. The transfer matrix method (TMM) [59] is then employed to calculate the multi‐state absorptivity of the generated RMMs and compare them with the target absorptivity spectral pair. Figure 3 displays the results of the inverse design of RMMs by CoSP.

The following conclusions can be drawn from Figure 3: (1) The spectrum descriptions generated by CoSP accurately depict the characteristics of the input spectral pair in two states, which strongly demonstrates that the spectrum encoder of CoSP has learned highly useful, high‐quality spectral representations and features. It also validates the effectiveness of contrastive multi‐state spectrum pretraining. (2) The RMM structures generated by CoSP possess spectral pairs essentially consistent with the targets, leading to the accurate and efficient inverse design of RMMs for the first time.

Additional inverse‐design examples spanning a broader variety of spectral pairs are provided in Section S6, further illustrating the accuracy and robustness of CoSP.

#### 4.2.3 Exploration of Hypothetical Spectra

In practical applications, RMMs are expected to show extreme optical response in desired wavelength bands. For instance, a high absorptivity in the visible wavelength band, a low absorptivity in the near‐infrared (IR) wavelength band, and again a high absorptivity in the mid‐IR wavelength band. Such complicated requirements lead to target optical spectra that mimic square‐wave functions. However, these square‐wave‐like spectra should be regarded as idealized design targets rather than exact responses that can be directly realized by practical materials. To further demonstrate the potential of CoSP beyond the test distribution, its design capacities for such extreme, hypothetical target spectra are investigated.

IR camouflage based on radiative RMMs is selected as an example [60], which enables temperature‐independent thermal radiance to evade IR detection by independently regulating multi‐band emissivity according to environmental temperatures [61]. Here, three IR wavelength bands are involved: infrared detectors typically operate in the 2–5 and 8–14 μ m bands [18], while the 5–8 μ m band (undetectable by IR sensors) is used for radiative cooling to help regulate the actual temperature [62]. As a result, at high environmental temperatures, the RMMs (**State M**) are expected to have low emissivity in both 2–5  and 8–14 μ m bands and high emissivity in the 5–8 μ m band for strong radiative cooling, all of which help reduce the total thermal radiance. Conversely, at low environmental temperatures, RMMs (**State I**) need high emissivity in both 2–5 and 8–14 μ m bands as well as low emissivity in the 5–8 μ m band for heat retention, leading to enhanced thermal radiance. With the above emissivity features, the total thermal radiance from RMM is deeply regulated and temperature‐independent for IR camouflage applications.

**FIGURE 4:**
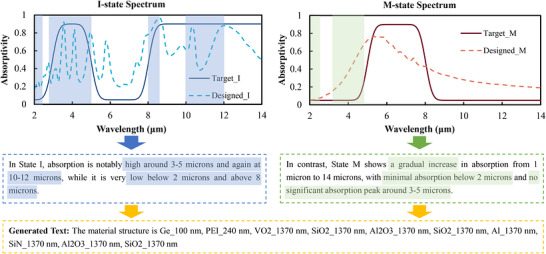
Inverse design results of CoSP for idealized IR‐camouflage applications.

In the experiment, the emissivity of the target spectral pair was set to either 0.9 or 0.05 at different wavelength bands. A Gaussian filter is then applied to smooth the square‐shaped design targets before inputting them into CoSP. As shown in Figure 4, though the target spectral pair represents an aggressively idealized, piecewise‐constant objective that is (almost) impossible in nature, CoSP still yields physically viable RMM candidates whose spectra track the targets while remaining paired with coherent natural‐language spectrum descriptions. This example therefore highlights the ability of CoSP to steer structured, physics‐conditioned generation toward demanding spectral programs that lie outside the smoothly varying spectra in the training distribution—a complementary capability to the large quantitative margins over adapted baselines reported in Table 2. In summary, CoSP narrows the gap between idealized multi‐band spectral objectives and practically realizable RMM designs.

### 4.3 Spectrum Representation Quality

CoSP is designed to learn spectrum embeddings that are simultaneously **state‐discriminative** and **sample‐consistent**: the encoder should (1) separate the two states of each spectral pair in the latent space, and (2) preserve meaningful similarity structure so that the spectral pair from the same device remains coherently related. These properties are evaluated on a subset of the test set via UMAP (Uniform Manifold Approximation and Projection) [63] visualization and cross‐state similarity analysis.

**FIGURE 5:**
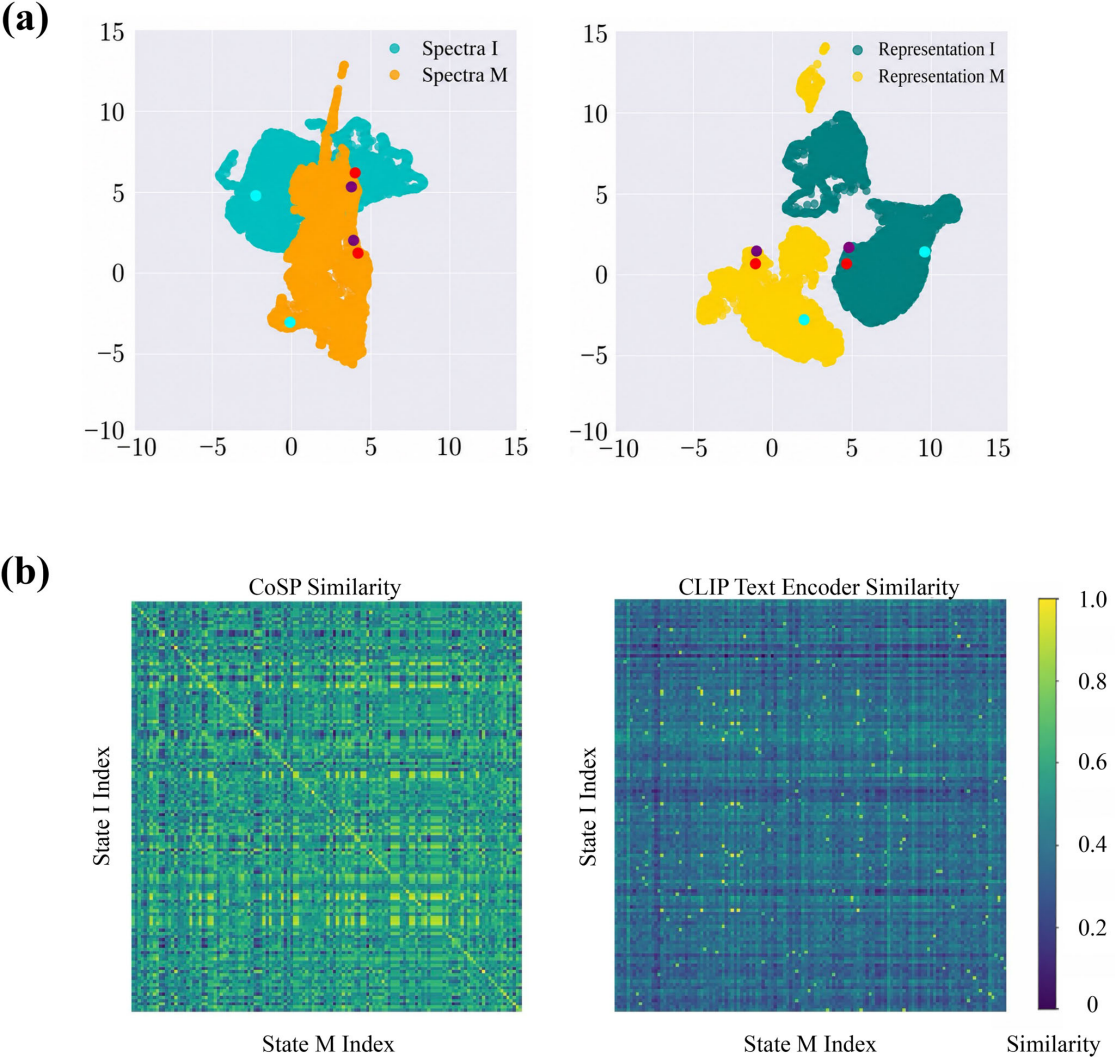
Evaluation of spectrum representation quality. (a) UMAP visualization of **raw spectra** (left) and **spectrum representations** learned by the CoSP spectrum encoder (right) on a subset of the test set. Three representative two‐state samples are highlighted as colored point pairs (red, purple, light blue), where the two points with the same color correspond to the spectral pair from the same metamaterial. (b) Cross‐state similarity matrices computed using the CoSP spectrum encoder and a CLIP text encoder pretrained in the same manner. CoSP exhibits pronounced diagonal dominance, indicating accurate cross‐state matching of spectra from the same sample.

Figure 5a visualizes the distributions of raw spectra (left) and the corresponding spectrum representations after UMAP dimensionality reduction (right). In the raw spectral space, State I and State M exhibit substantial overlapping, indicating that direct discrimination based on raw absorptivity spectra is intrinsically ambiguous under multi‐state variations. In contrast, the learned representations form two clearly separated clusters without overlapping, demonstrating that the spectrum encoder extracts compact, state‐informative features. Furthermore, three representative RMMs are highlighted as point pairs with different colors (red, purple, and light blue), where each color denotes one sample and the two points correspond to its two‐state spectra. Notably, the relative geometry is largely preserved: RMMs that are close in the raw spectrum embedding (e.g., the red and purple pairs) remain close in the latent embedding, whereas a more distant RMM (light blue) maintains a larger separation. This result indicates that the encoder enhances state separability while retaining intrinsic neighborhood relations rather than arbitrarily distorting the data manifold.

To further verify cross‐state **sample identity** matching, a similarity matrix is constructed between State I and State M representations. Given n test samples, $s_{k}^{I}$ and $s_{k}^{M}$ are obtained for k=1,…,n, forming $S^{I}={\left[s_{1}^{I},\text{…},s_{n}^{I}\right]}^{⊤}$ and $S^{M}={\left[s_{1}^{M},\text{…},s_{n}^{M}\right]}^{⊤}$, and then computing

(5) $$X=S^{I}\cdot {\left(S^{M}\right)}^{⊤},$$

where $X_{m,n}$ measures the similarity between the State I representation of metamaterial m and the State M representation of metamaterial n. Ideally, the largest entry in each row/column should lie on the diagonal with correct cross‐state pairing. Figure 5b shows that CoSP yields a strongly diagonal‐dominant similarity pattern, whereas a CLIP text encoder (pretrained in the same manner) does not exhibit comparable diagonal contrast. Together, these results confirm that CoSP learns high‐quality multi‐state spectrum representations that both separate states and reliably associate corresponding spectra between the two states of the same sample, providing a robust foundation for downstream inverse design of RMMs.

### 4.4 Ablation Study

**FIGURE 6:**
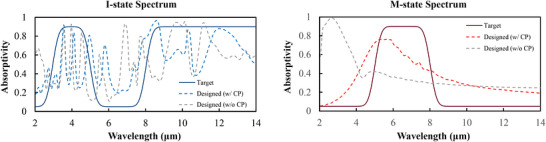
Ablation study on the effect of contrastive multi‐state spectrum pretrain. CoSP achieves an MSE of **0.152**, while the variant without contrastive spectrum pretrain yields **0.328**.

To clarify the role of contrastive multi‐state spectrum pretraining, an ablated variant of the full framework is constructed by removing the contrastive pretraining stage (Figure 6). It is evident that, for the same design objective, CoSP without contrastive multi‐state spectrum pretraining yields poor results. Furthermore, the designed spectra deviate significantly from the target spectra with much higher MSE. This comparison clearly demonstrates the effectiveness and necessity of contrastive multi‐state spectrum pretraining.

## 5 Conclusion

In this paper, CoSP, a novel inverse design model that leverages contrastive multi‐state spectrum pretraining and LLMs, is introduced to achieve the accurate and efficient inverse design of RMMs for the first time. CoSP encompasses all capabilities of existing models. Experimental results demonstrate that CoSP not only excels in designing spectral pairs within the test set, but also achieves precise approximation of spectra corresponding to hypothetical square‐wave functions that are otherwise unattainable by natural materials. As a versatile tool, CoSP not only streamlines the development of RMMs for thermal management, optical computation, and telecommunications applications but also establishes a new paradigm for AI‐for‐Science in the discovery of smart photonic materials. Beyond its immediate implications for photonics, the CoSP framework can be seamlessly extended to other reconfigurable devices and multi‐physics design challenges, such as 2D metasurfaces and 3D metamaterials, paving the way for a new generation of intelligent inverse‐design methodologies.

## Author Contributions

Shujie Yang: Conceptualization, Methodology, Coding, Writing – original draft. Yuqi Zhang: Conceptualization, Methodology, Coding, Writing – original draft. Xuzhe Zhao: Visualization, Writing – review & editing. Yansong Tang: Supervision, Writing – review & editing. Kaichen Dong: Conceptualization, Supervision, Project administration, Funding acquisition, Writing – review & editing.

## Conflicts of Interest

The authors declare no conflicts of interest.

## Supporting information


**Supporting File**: advs76917‐sup‐0001‐SuppMat.pdf.

## Data Availability

The code and data supporting the findings of this study are available at https://github.com/YangSJ2019/CoSP.

## References

[1] R. A. Shelby , D. R. Smith , and S. Schultz , “Experimental Verification of a Negative Index of Refraction,” Science 292, no. 5514 (2001): 77–79. 11292865 10.1126/science.1058847

[2] N. Segal , S. Keren‐Zur , N. Hendler , and T. Ellenbogen , “Controlling Light With Metamaterial‐Based Nonlinear Photonic Crystals,” Nature Photonics 9, no. 3 (2015): 180–184.

[3] S. Zhang , W. Fan , N. Panoiu , K. Malloy , R. Osgood , and S. Brueck , “Experimental Demonstration of Near‐Infrared Negative‐Index Metamaterials,” Physical Review Letters 95, no. 13 (2005): 137404. 16197179 10.1103/PhysRevLett.95.137404

[4] D. R. Smith , J. B. Pendry , and M. C. Wiltshire , “Metamaterials and Negative Refractive Index,” Science 305, no. 5685 (2004): 788–792. 15297655 10.1126/science.1096796

[5] L. Zhang , X. Q. Chen , S. Liu , et al., “Space‐Time‐Coding Digital Metasurfaces,” Nature Communications 9, no. 1 (2018): 4334. 10.1038/s41467-018-06802-0 PMC6194064 30337522

[6] N. I. Zheludev and Y. S. Kivshar , “From Metamaterials to Metadevices,” Nature Materials 11, no. 11 (2012): 917–924. 23089997 10.1038/nmat3431

[7] K. V. Sreekanth , Y. Alapan , M. ElKabbash , et al., “Extreme Sensitivity Biosensing Platform Based on Hyperbolic Metamaterials,” Nature Materials 15, no. 6 (2016): 621–627. 27019384 10.1038/nmat4609 PMC4959915

[8] A. P. Raman , M. A. Anoma , L. Zhu , E. Rephaeli , and S. Fan , “Passive Radiative Cooling Below Ambient Air Temperature Under Direct Sunlight,” Nature 515, no. 7528 (2014): 540–544. 25428501 10.1038/nature13883

[9] W. Ma , Z. Liu , Z. A. Kudyshev , A. Boltasseva , W. Cai , and Y. Liu , “Deep Learning for the Design of Photonic Structures,” Nature Photonics 15, no. 2 (2021): 77–90.

[10] A. Sakurai , K. Yada , T. Simomura , et al., “Ultranarrow‐Band Wavelength‐Selective Thermal Emission With Aperiodic Multilayered Metamaterials Designed by Bayesian Optimization,” ACS Central Science 5, no. 2 (2019): 319–326. 30834320 10.1021/acscentsci.8b00802 PMC6396383

[11] H. Wang , Z. Zheng , C. Ji , and L. J. Guo , “Automated Multi‐Layer Optical Design via Deep Reinforcement Learning,” Machine Learning: Science and Technology 2, no. 2 (2021): 025013.

[12] J. T. Overvelde , J. C. Weaver , C. Hoberman , and K. Bertoldi , “Rational Design of Reconfigurable Prismatic Architected Materials,” Nature 541, no. 7637 (2017): 347–352. 28102254 10.1038/nature20824

[13] J. P. Turpin , J. A. Bossard , K. L. Morgan , D. H. Werner , and P. L. Werner , “Reconfigurable and Tunable Metamaterials: A Review of the Theory and Applications,” International Journal of Antennas and Propagation 2014, no. 1 (2014): 429837.

[14] T. Ma , H. Wang , and L. J. Guo , “Optogpt: A Foundation Model for Inverse Design in Optical Multilayer Thin Film Structures,” Opto‐Electronic Advances 7, no. 7 (2024): 240062, 1.

[15] R. Unni , K. Yao , and Y. Zheng , “Deep Convolutional Mixture Density Network for Inverse Design of Layered Photonic Structures,” ACS Photonics 7, no. 10 (2020): 2703–2712. 38031541 10.1021/acsphotonics.0c00630 PMC10686261

[16] W. Chen , Y. Gao , Y. Li , et al., “Broadband Solar Metamaterial Absorbers Empowered by Transformer‐Based Deep Learning,” Advanced Science 10, no. 13 (2023): 2206718. 36852630 10.1002/advs.202206718 PMC10161039

[17] S. Yu , P. Zhou , W. Xi , et al., “General Deep Learning Framework for Emissivity Engineering,” Light: Science & Applications 12, no. 1 (2023): 291. 10.1038/s41377-023-01341-w PMC10697983 38052800

[18] W. Xi , Y.‐J. Lee , S. Yu , et al., “Ultrahigh‐Efficient Material Informatics Inverse Design of Thermal Metamaterials for Visible‐Infrared‐Compatible Camouflage,” Nature Communications 14, no. 1 (2023): 4694. 10.1038/s41467-023-40350-6 PMC10403604 37542047

[19] Y. Huang , S. Wu , S. Zhao , et al., “A Novel Liquid Flow Electrochromic Smart Window for All‐Year‐Round Dynamic Photothermal Regulation,” Energy & Environmental Science 18 (2025): 1824–1834.

[20] K. Tang , K. Dong , J. Li , et al., “Temperature‐Adaptive Radiative Coating for All‐Season Household Thermal Regulation,” Science 374, no. 6574 (2021): 1504–1509. 34914515 10.1126/science.abf7136

[21] M. Y. Shalaginov , S. An , Y. Zhang , et al., “Reconfigurable All‐Dielectric Metalens With Diffraction‐Limited Performance,” Nature Communications 12, no. 1 (2021): 1225. 10.1038/s41467-021-21440-9 PMC7900249 33619270

[22] P. C. Wu , R. A. Pala , G. Kafaie Shirmanesh , et al., “Dynamic Beam Steering With All‐Dielectric Electro‐Optic III–V Multiple‐Quantum‐Well Metasurfaces,” Nature Communications 10, no. 1 (2019): 3654. 10.1038/s41467-019-11598-8 PMC6692380 31409790

[23] C. Sui , J. Pu , T.‐H. Chen , et al., “Dynamic Electrochromism for All‐Season Radiative Thermoregulation,” Nature Sustainability 6, no. 4 (2023): 428–437.

[24] J.‐Y. Ou , E. Plum , J. Zhang , and N. I. Zheludev , “An Electromechanically Reconfigurable Plasmonic Metamaterial Operating in the Near‐Infrared,” Nature Nanotechnology 8, no. 4 (2013): 252–255. 10.1038/nnano.2013.25 23503091

[25] X. A. Zhang , S. Yu , B. Xu , et al., “Dynamic Gating of Infrared Radiation in a Textile,” Science 363, no. 6427 (2019): 619–623. 30733415 10.1126/science.aau1217

[26] Q. Zhang , Y. Jiang , L. Chen , et al., “Ultra‐Compliant and Tough Thermochromic Polymer for Self‐Regulated Smart Windows,” Advanced Functional Materials 31, no. 18 (2021): 2100686.

[27] K. Liu , S. Lee , S. Yang , O. Delaire , and J. Wu , “Recent Progresses on Physics and Applications of Vanadium Dioxide,” Materials Today 21, no. 8 (2018): 875–896.

[28] S. Wang , T. Jiang , Y. Meng , R. Yang , G. Tan , and Y. Long , “Scalable Thermochromic Smart Windows With Passive Radiative Cooling Regulation,” Science 374, no. 6574 (2021): 1501–1504. 34914526 10.1126/science.abg0291

[29] S. Chandra , D. Franklin , J. Cozart , A. Safaei , and D. Chanda , “Adaptive Multispectral Infrared Camouflage,” ACS Photonics 5, no. 11 (2018): 4513–4519.

[30] P. Khosla , P. Teterwak , C. Wang , et al., “Supervised Contrastive Learning,” Advances in Neural Information Processing Systems 33 (2020): 18661–18673.

[31] C.‐Y. Chuang , J. Robinson , Y.‐C. Lin , A. Torralba , and S. Jegelka , “Debiased Contrastive Learning,” Advances in Neural Information Processing Systems 33 (2020): 8765–8775.

[32] T. Gao , X. Yao , and D. Chen , “Simcse: Simple Contrastive Learning of Sentence Embeddings,” preprint, arXiv, April 18, 2021, 10.48550/arXiv.2104.08821.

[33] A. V. D. Oord , Y. Li , and O. Vinyals , “Representation Learning With Contrastive Predictive Coding,” preprint, arXiv, July 10, 2018, 10.48550/arXiv.1807.03748.

[34] T. Chen , S. Kornblith , M. Norouzi , and G. Hinton , “A Simple Framework for Contrastive Learning of Visual Representations,” in International Conference on Machine Learning (PMLR, 2020), 1597–1607.

[35] K. He , H. Fan , Y. Wu , S. Xie , and R. Girshick , “Momentum Contrast for Unsupervised Visual Representation Learning,” in Proceedings of the IEEE/CVF Conference on Computer Vision and Pattern Recognition (IEEE, 2020), 9729–9738.

[36] A. Radford , J. W. Kim , C. Hallacy , et al., “Learning Transferable Visual Models From Natural Language Supervision,” in International Conference on Machine Learning (PMLR, 2021), 8748–8763.

[37] A. Radford , J. Wu , R. Child , D. Luan , D. Amodei , and I. Sutskever , “Language Models are Unsupervised Multitask Learners,” OpenAI Technical Report, OpenAI, San Francisco, CA, USA, 2019, pp, (2019): 1–24, https://cdn.openai.com/better‐language‐models/language_models_are_unsupervised_multitask_learners.pdf.

[38] T. Brown , B. Mann , N. Ryder , et al., “Language Models are Few‐Shot Learners,” Advances in Neural Information Processing Systems 33 (2020): 1877–1901.

[39] J. Achiam , S. Adler , S. Agarwal , et al., “Gpt‐4 Technical Report,” preprint, arXiv, March 24, 2024, 10.48550/arXiv.2303.08774.

[40] A. Chowdhery , S. Narang , J. Devlin , et al., “Palm: Scaling Language Modeling With Pathways,” Journal of Machine Learning Research 24, no. 240 (2023): 1–113.

[41] H. Touvron , T. Lavril , G. Izacard , et al., “Llama: Open and Efficient Foundation Language Models,” preprint, arXiv, February 27, 2023, 10.48550/arXiv.2302.13971.

[42] P. Lewis , E. Perez , A. Piktus , et al., “Retrieval‐Augmented Generation for Knowledge‐Intensive NLP Tasks,” Advances in Neural Information Processing Systems 33 (2020): 9459–9474.

[43] Q. Jin , Y. Yang , Q. Chen , and Z. Lu , “Genegpt: Augmenting Large Language Models With Domain Tools for Improved Access to Biomedical Information,” Bioinformatics 40, no. 2 (2024): btae075. 38341654 10.1093/bioinformatics/btae075 PMC10904143

[44] S. Miret and N. Krishnan , “Are LLMs Ready for Real‐World Materials Discovery?” preprint, arXiv, September 25, 2024, 10.48550/arXiv.2402.05200.

[45] A. M. Bran , S. Cox , O. Schilter , C. Baldassari , A. D. White , and P. Schwaller , “Augmenting Large Language Models With Chemistry Tools,” Nature Machine Intelligence 6 (2024): 525–535. 10.1038/s42256-024-00832-8 PMC11116106 38799228

[46] D. Zhang , W. Liu , Q. Tan , et al., “Chemllm: A Chemical Large Language Model,” preprint, arXiv, April, 25, 2024, 10.48550/arXiv.2402.06852.

[47] D. A. Boiko , R. MacKnight , B. Kline , and G. Gomes , “Autonomous Chemical Research With Large Language Models,” Nature 624, no. 7992 (2023): 570–578. 38123806 10.1038/s41586-023-06792-0 PMC10733136

[48] H. Abdine , M. Chatzianastasis , C. Bouyioukos , and M. Vazirgiannis , “Prot2text: Multimodal Protein's Function Generation With GNNs and Transaformers,” Proceedings of the AAAI Conference on Artificial Intelligence 38, no. 10 (2024): 10757–10765.

[49] M. Kim , H. Park , and J. Shin , “Nanophotonic Device Design Based on Large Language Models: Multilayer and Metasurface Examples,” Nanophotonics 14 (2025): 1273–1282. 40290288 10.1515/nanoph-2024-0674 PMC12019936

[50] D. Lu , Y. Deng , J. M. Malof , and W. J. Padilla , “Learning Electromagnetic Metamaterial Physics With ChatGPT,” IEEE Access 13 (2025): 51513–51526.

[51] H. Zhang , L. Kang , S. D. Campbell , and D. H. Werner , “Chat to Chip: Large Language Model Based Design of Arbitrarily Shaped Metasurfaces,” Nanophotonics 14 (2025): 3625–3633. 41209412 10.1515/nanoph-2025-0343 PMC12592791

[52] A. Vaswani , N. Shazeer , N. Parmar , et al., “Attention is All You Need,” Advances in Neural Information Processing Systems, 30 (2017): 5998–6008, https://proceedings.neurips.cc/paper_files/paper/2017/hash/3f5ee243547dee91fbd053c1c4a845aa‐Abstract.html.

[53] D. Fu , K. Liu , T. Tao , et al., “Comprehensive Study of the Metal‐Insulator Transition in Pulsed Laser Deposited Epitaxial ${\mathrm{VO}}_{2}$ Thin Films,” Journal of Applied Physics 113, no. 4 (2013): 043707.

[54] T. Guo , Z. Zhang , Z. Lin , et al., “Durable and Programmable Ultrafast Nanophotonic Matrix of Spectral Pixels,” Nature Nanotechnology 19, no. 11 (2024): 1635–1643. 10.1038/s41565-024-01756-5 PMC11567887 39134690

[55] Y. Zhou , Z. Qin , Z. Liang , et al., “Ultra‐Broadband Metamaterial Absorbers From Long to Very Long Infrared Regime,” Light: Science & Applications 10, no. 1 (2021): 138. 10.1038/s41377-021-00577-8 PMC8257711 34226489

[56] J. Li , K. Dong , T. Zhang , et al., “Printable, Emissivity‐Adaptive and Albedo‐Optimized Covering for Year‐Round Energy Saving,” Joule 7, no. 11 (2023): 2552–2567.

[57] M. Mirza and S. Osindero , “Conditional Generative Adversarial Nets,” preprint, arXiv, November 06, 2014, 10.48550/arXiv.1411.1784.

[58] K. Sohn , H. Lee , and X. Yan , “Learning Structured Output Representation Using Deep Conditional Generative Models,” Advances in Neural Information Processing Systems 28 (2015): 3483–3491, https://proceedings.neurips.cc/paper/2015/hash/8d55a249e6baa5c06772297520da2051‐Abstract.html.

[59] J.‐H. Song , P. Lalanne , M.‐K. Seo , and M. L. Brongersma , “Transfer Matrix Method‐Compatible Model for Metamaterial Stacks,” ACS Photonics 10, no. 8 (2023): 2948–2954.

[60] H. Zhu , Q. Li , C. Tao , et al., “Multispectral Camouflage for Infrared, Visible, Lasers and Microwave With Radiative Cooling,” Nature Communications 12, no. 1 (2021): 1805. 10.1038/s41467-021-22051-0 PMC7985314 33753740

[61] K. Tang , X. Wang , K. Dong , et al., “A Thermal Radiation Modulation Platform by Emissivity Engineering With Graded Metal–Insulator Transition,” Advanced Materials 32, no. 36 (2020): 1907071. 10.1002/adma.201907071 32700403

[62] B. Qin , Y. Zhu , Y. Zhou , M. Qiu , and Q. Li , “Whole‐Infrared‐Band Camouflage With Dual‐Band Radiative Heat Dissipation,” Light: Science & Applications 12, no. 1 (2023): 246. 10.1038/s41377-023-01287-z PMC10550919 37794015

[63] L. Mcinnes and J. Healy , “UMAP: Uniform Manifold Approximation and Projection for Dimension Reduction,” Journal of Open Source Software 3, no. 29 (2018): 861.

