# Supplementary material for: CoSP: Reconfigurable Metamaterial Inverse Design via Contrastive Pretrained Large Language Model
Source: Adv Sci (Weinh). 2026 Aug 11;13(61):e76917. doi: 10.1002/advs.76917 (PMC13460417; doi:10.1002/advs.76917)
Supplement: Supplementary file 1 [file ADVS-13-e76917-s001.pdf]

Supporting Information for:
CoSP: Reconfigurable Metamaterial Inverse Design via Contrastive Pretrained Large Language Model

Shujie Yang[1,2,3,†] Yuqi Zhang[1,2,3,†] Xuzhe Zhao[1,2,3] Yansong Tang[1,] Kaichen Dong[1,2,3,*]*

[1]Institute of Data and Information, Tsinghua Shenzhen International Graduate School, Tsinghua University, Shenzhen, Guangdong 518055, China
[2]Center of Double Helix, Tsinghua Shenzhen International Graduate School, Tsinghua University, Shenzhen, Guangdong 518055, China
[3]Intelligent Passive Thermal Control Center, Research Institute of Tsinghua University in Shenzhen, Shenzhen, Guangdong 518057, China
[†]These authors contributed equally to this work.
[*]Corresponding authors. Email: tang.yansong@sz.tsinghua.edu.cn; dkc22@sz.tsinghua.edu.cn

S1 Further Explanation of CoSP's Advantages

To provide a clearer explanation of CoSP's advantages over previous inverse design methods in the **material** and **objective** dimensions, this section offers a detailed elaboration. **Figure S1** illustrates CoSP's superiority in these two dimensions: In the material dimension, most existing works [1, 2, 3] require predefined layer numbers and predefined material assignments for each layer (e.g., Layer 1: Material A, Layer 2: Material B...), severely limiting flexibility. In contrast, CoSP exhibits high freedom in both structural configurations and material sets: layer thicknesses and the number of layers can be freely designed, while materials for each layer can be freely selected from a large material library. This represents a significant advancement in design flexibility. In the objective dimension, most existing works [1, 3, 4] require training with a specific target spectrum as input and a predefined structure with materials. If the target spectra or the predefined structures (with materials) change, the entire model must be retrained, demonstrating poor transferability. In contrast, CoSP is a general design framework capable of designing from "multi-state spectra" without retraining, as validated in the main text experimental section. In other words, CoSP, trained only once, can handle versatile tasks, showcasing enhanced transferability.

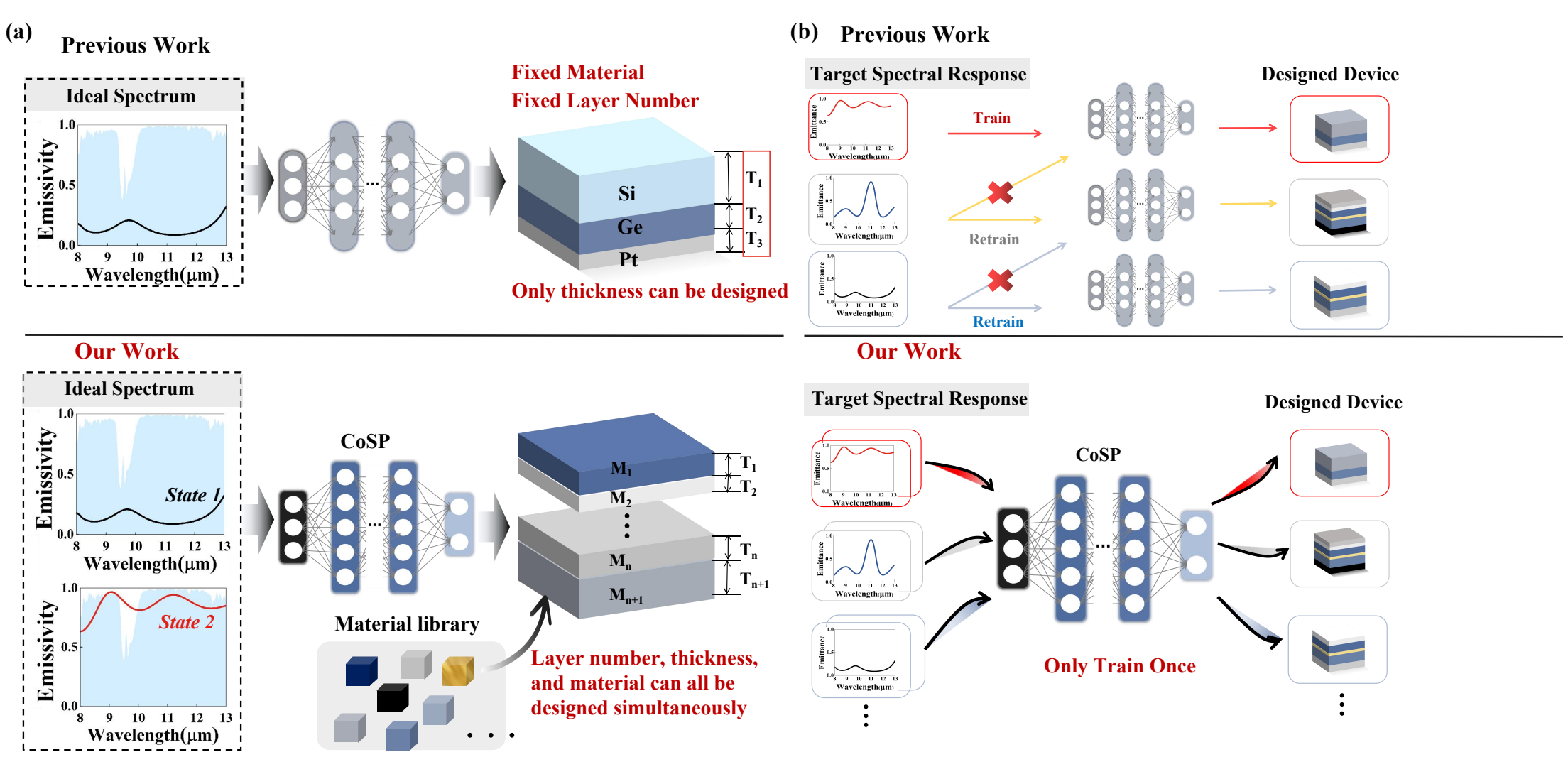

Figure S1: **Schematic figure of CoSP's advantages over previous work on (a) material and (b) objective dimension.**

S2 Ablation of Spectrum Description

Due to the one-to-many mapping of spectra to materials, spectrum description is incorporated as an additional training objective to ensure training quality and enhance the model's generalization capabilities. To evaluate the effectiveness of incorporating spectrum description, material structure generation training is conducted with and without it. **Figure S2** illustrates the loss on the training and validation sets with and without the inclusion of spectrum description. It is evident that the inclusion of spectrum description as a training objective results in a more rapid decrease in loss and a lower overall loss on the validation set. This indicates that incorporating spectrum descriptions significantly improves the model's performance and convergence speed.

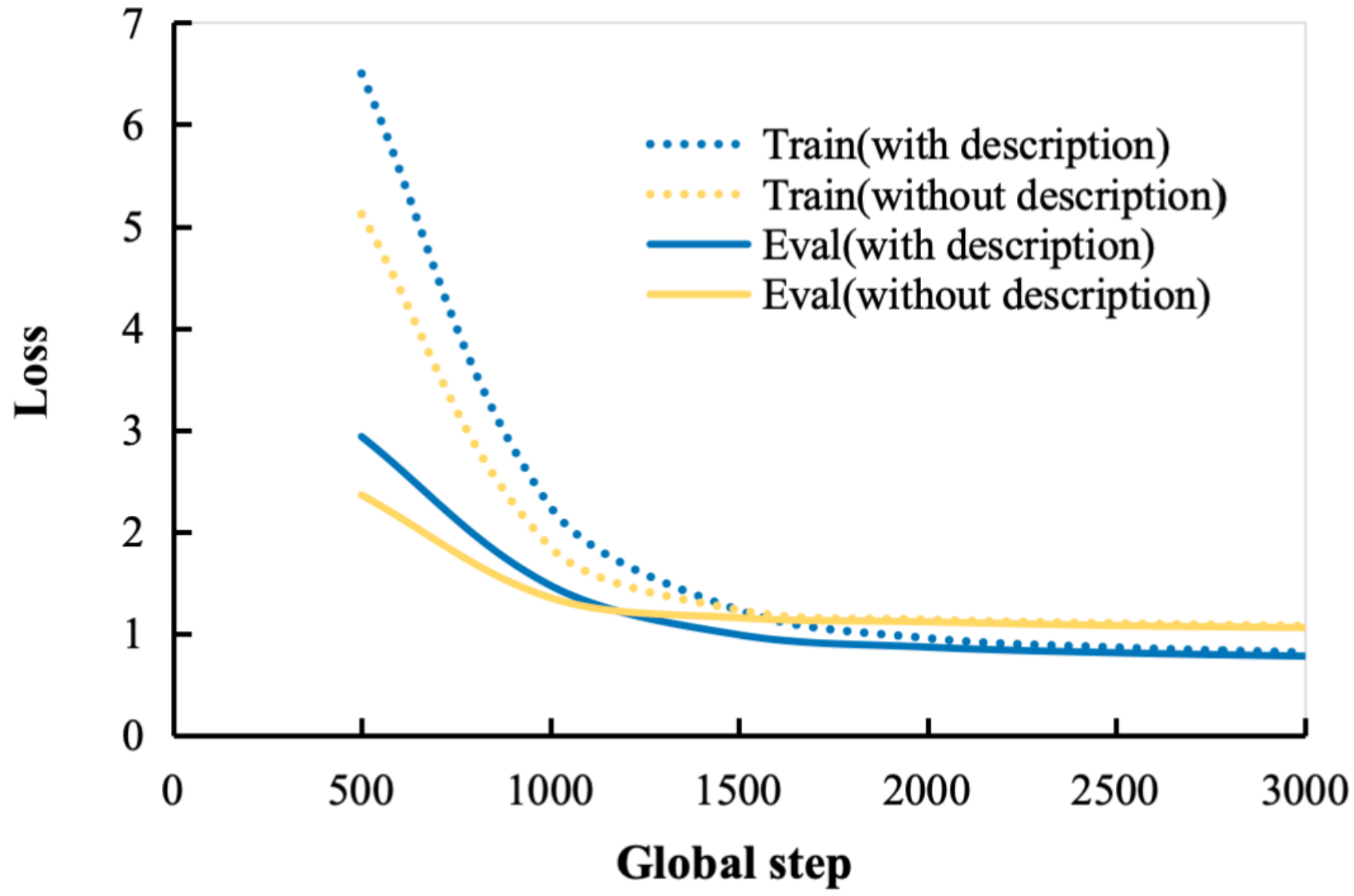

Figure S2: **Loss curves on the training and validation sets when CoSP generates or does not generate spectrum descriptions.**

S3 Data Generation

S3.1 Transfer Matrix Method

The reflectivity or absorptivity spectra used in this model are generated by the Transfer Matrix Method (TMM). TMM is a mathematical analysis model widely used in optics, electromagnetics, acoustics, and quantum mechanics to describe wave propagation using matrix operations [5]. Taking optics as an example, TMM can easily solve the reflectivity and absorptivity of one-dimensional photonic crystals or multilayer metamaterials. The specific solution process can be divided into the following steps: first, the multilayer metamaterial is modeled layer by layer, and boundary conditions are set, which means inputting the physical properties of different layers (such as refractive indices) and determining the interlayer relationship of wave propagation. Then, based on the interlayer modeling, establish the transmission matrix corresponding to the refractive index for each layer. Assume that the thickness of a dielectric layer is t, the wave number is k, and the refractive index of the material used in the layer is n. The optical response of each multilayer structure was calculated using the coherent TMM implemented by the open-source package [6] . The complex refractive index of layer i is

$$\tilde{n}_i(\lambda) = n_i(\lambda) + ik_i(\lambda) \tag{S1}$$

and the propagation angle in each layer is determined by Snell's law,

$$\tilde{n}_0 \sin\theta_0 = \tilde{n}_i \sin\theta_i \tag{S2}$$

The Fresnel amplitude coefficients at the interface between media i and $i+1$ are

$$r_{i,i+1} = \frac{\tilde{n}_i \cos\theta_i - \tilde{n}_{i+1} \cos\theta_{i+1}}{\tilde{n}_i \cos\theta_i + \tilde{n}_{i+1} \cos\theta_{i+1}} \tag{S3a}$$

$$t_{i,i+1} = \frac{2\tilde{n}_i \cos\theta_i}{\tilde{n}_i \cos\theta_i + \tilde{n}_{i+1} \cos\theta_{i+1}} \tag{S3b}$$

The propagation matrices are defined as

$$\mathbf{P}_i = \begin{pmatrix} e^{-i\delta_i} & 0 \\ 0 & e^{i\delta_i} \end{pmatrix} \tag{S4a}$$

$$\delta_i = \frac{2\pi}{\lambda} \tilde{n}_i d_i \cos\theta_i \tag{S4b}$$

and the interface matrices are defined as

$$\mathbf{I}_{i,i+1} = \frac{1}{t_{i,i+1}} \begin{pmatrix} 1 & r_{i,i+1} \\ r_{i,i+1} & 1 \end{pmatrix} \tag{S5}$$

Accordingly, the total transfer matrix is

$$\mathbf{M}_{\text{total}} = \mathbf{I}_{0,1}\mathbf{P}_1\mathbf{I}_{1,2}\mathbf{P}_2 \cdots \mathbf{P}_N\mathbf{I}_{N,N+1} \tag{S6}$$

Here, $\mathbf{P}_i$ describes phase accumulation and attenuation within layer i, whereas $\mathbf{I}_{i,i+1}$ accounts for Fresnel reflection and transmission at the interface between adjacent media. With the incident amplitude normalized to unity and no wave incident from the exit side,

$$\begin{pmatrix} 1 \\ r \end{pmatrix} = \mathbf{M}_{\text{total}} \begin{pmatrix} t \\ 0 \end{pmatrix} \tag{S7}$$

If it is defined that

$$\mathbf{M}_{\text{total}} = \begin{pmatrix} M_{11} & M_{12} \\ M_{21} & M_{22} \end{pmatrix} \tag{S8}$$

the total complex reflection and transmission amplitude coefficients are

$$r = \frac{M_{21}}{M_{11}} \tag{S9a}$$

$$t = \frac{1}{M_{11}} \tag{S9b}$$

The reflectivity, transmissivity, and absorptivity are then calculated as

$$R = |r|^2 \tag{S10a}$$

$$T = |t|^2 \frac{\mathrm{Re}(\tilde{n}_{N+1} \cos\theta_{N+1})}{\mathrm{Re}(\tilde{n}_0 \cos\theta_0)} \tag{S10b}$$

$$A = 1 - R - T \tag{S10c}$$

S3.2 Data Generation Details

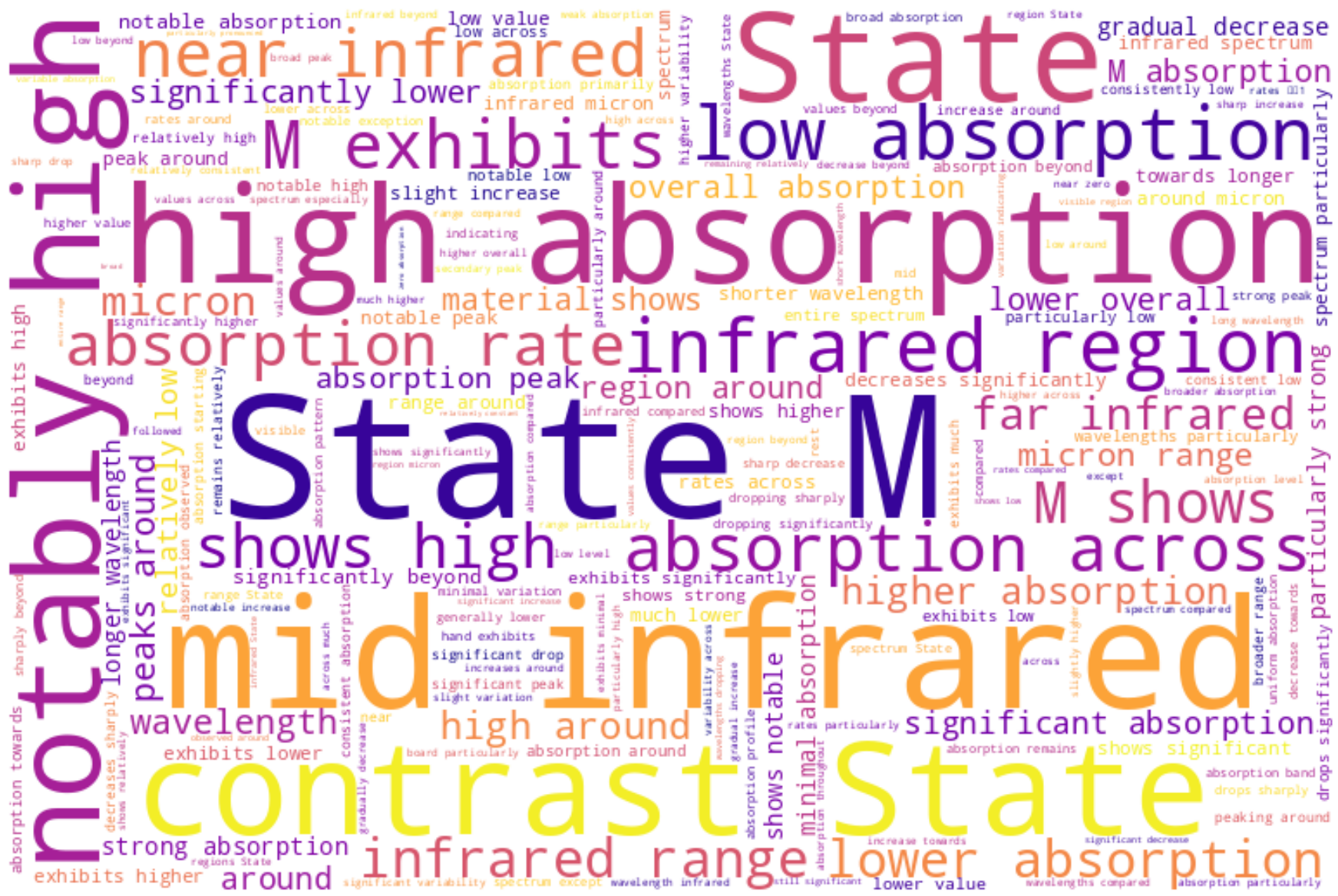

Figure S3: **Word cloud visualization of spectrum descriptions in the dataset.**

To train CoSP, material compositions and thicknesses of all layers within the RMM are randomly selected from a library of 13 materials, including vanadium dioxide for multi-state switching, to form combinations. According to the practical limitations by micro-fabrication facilities, the number of layers and thicknesses are randomized, with the number of layers ranging from a minimum of 5 to a maximum of 12, and thicknesses ranging from a minimum of 30 nm to a maximum of 5,000 nm. The TMM is then used to calculate the 0.4-14 µm ultra-broadband absorptivity of both State I and State M of these RMMs. A total of 120,000 candidate samples were initially generated. After the removal of anomalous records, with 98,628 used for training, 9,870 used for validation and 10,000 used for testing. For the spectrum descriptions, Qwen2.5 [7] is utilized for generation. **Figure S3** shows the word cloud of spectrum descriptions in the training set, where larger words indicate higher frequencies of occurrence. For every RMM, reflectivity (R), and transmissivity (T) are computed using the coherent TMM, and absorptivity (A) is calculated by $A(\lambda) = 1 - R(\lambda) - T(\lambda)$. For reciprocal RMMs under the same wavelength, incidence angle, and polarization, the directional spectral emissivity equals the absorptivity according to Kirchhoff's law. Accordingly, absorptivity is used as the optimization target, while reflectivity and transmissivity are retained to characterize the complete optical response. All multilayer spectra are calculated for a free-standing air/multilayer/air configuration over 0.4–14 μm using 100 wavelength points. The calculations are performed at normal incidence ($\theta = 0°$) with s-polarization.
A comprehensive material library is established consisting of 13 distinct materials, which are as follows: Germanium (Ge) [8], Vanadium Dioxide (VO_2) [9], Barium Fluoride (BaF_2) [10], Zinc Sulfide (ZnS) [11, 12], Aluminum Oxide (Al_2O_3) [13], Polyethylene (PE) [14, 15], Polydimethylsiloxane (PDMS) [16, 17], Silicon Dioxide (SiO_2) [18, 19], Polyetherimide (PEI) [16, 17], Silicon Nitride (SiN) [18, 20], Titanium Dioxide (TiO_2) [21], Aluminum (Al) [22], and Tungsten (W) [23, 24].

{"role": "system", "content": "You are Qwen, an advanced assistant and knowledgeable physicist. Your expertise includes spectral analysis and material sciences."**},**
{“role”: “user”, “content”: (
"The material exhibits varying absorption rates at 100 distinct points over the infrared spectrum, "
"ranging from 0.4 to 14 microns. Each point in this range corresponds directly to the absorption rate "
"of the material at that specific wavelength. As a physicist specializing in material properties, "
"your task is to analyze absorption characteristics."
f"For state I, the absorption rates are as follows: {Absorption_I}. "
f"For state M, the absorption rates are as follows: {Absorption_M}. "
"Based on this data, please provide a concise description of the key features of these absorption rates, "
"indicating in which spectral ranges the absorption is notably high or low for each state. "
"Respond with just one or two sentences in English.”**)**
}

Figure S4: **Structured prompt used for generating spectrum descriptions.**

Multi-phase spectrum	Spectrum Description	Material Structure
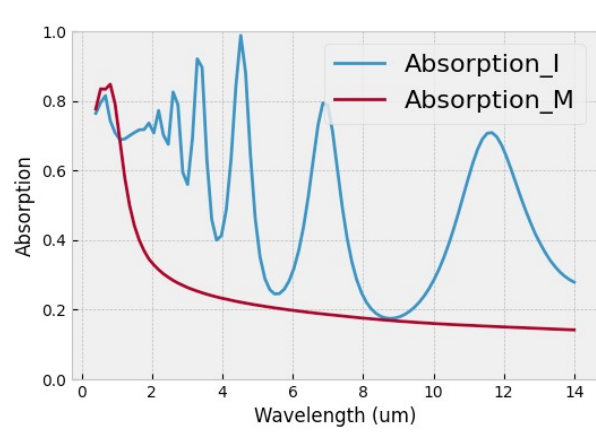	State I shows significant absorption in the mid-infrared range (3-5 microns) with peaks around 3 and 4 microns, while State M exhibits high absorption only up to 2 microns and very low absorption in the mid-infrared to far-infrared range, with a sharp drop after 2 microns.	VO_2_1930 nm, Al_4530 nm, BaF_2_290 nm, TiO_2_2300 nm, Al_980 nm, BaF2_3800 nm, PDMS_4180 nm, Ge_3370 nm
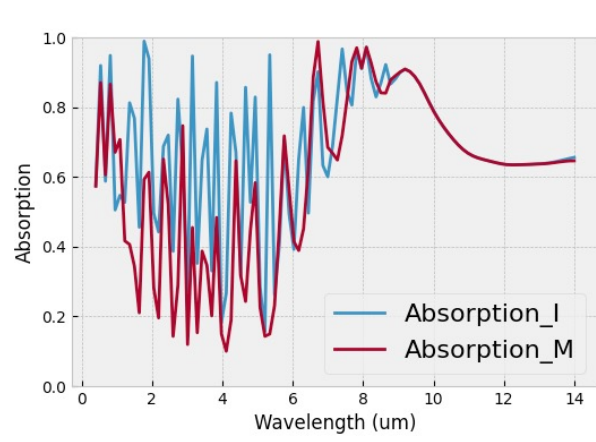	In State I, the material shows strong absorption in the mid-infrared region (3-5 microns) and a gradual decrease in absorption towards longer wavelengths, while in State M, absorption is notably lower in the visible and near-infrared regions but increases significantly in the mid-infrared region, particularly around 5 microns.	SiN_3920 nm, PDMS_4960 nm, VO_2_1600 nm, BaF_2_4490 nm, Al_2O_3_4200 nm, Al_4930 nm, SiO_2_1940 nm, PE_410 nm, Al_2540 nm
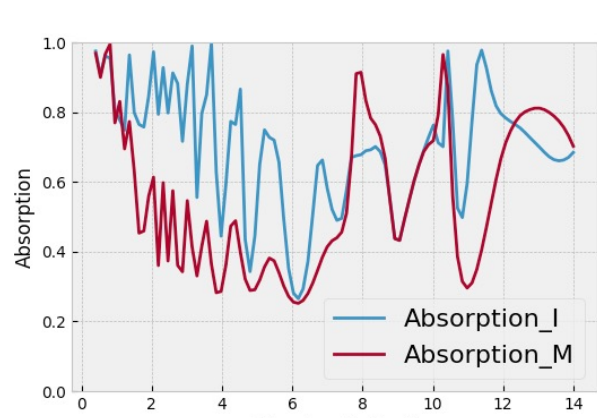	In state I, the material shows high absorption rates (close to 1) in the range of 0.4 to 2 microns and a gradual decrease to lower values around 0.4-0.8 in the mid-infrared range, with minimal absorption above 6 microns. In contrast, state M exhibits high absorption primarily in the near-infrared region below 3 microns, with significantly lower absorption rates across most of the mid-infrared spectrum, except for a slight increase around 8 to 10 microns.	SiO_2_2960 nm, PE_3570 nm, VO_2_1830 nm, SiO_2_3250 nm, W_750 nm

Figure S5: **Three examples of complete samples in the dataset.**

Each layer is assigned a thickness ranging from 30 to 5,000 nm. Subsequently, the TMM is employed to calculate the absorptivity of these layered structures across a wavelength spectrum of 0.4 to 14 µm. Utilizing this methodology, a total of 120,000 candidate samples were initially generated. After the removal of anomalous records, with 98,628 used for training, 9,870 used for validation and 10,000 used for testing. Following the data collection, Qwen2.5 is utilized to generate spectrum descriptions corresponding to the spectrum data. A structured instruction is established for Qwen, as shown in **Figure S4**. This marks the completion of the data generation process. In **Figure S5**, several detailed examples are presented to illustrate the contents included in the dataset.

S3.3 Transmissivity Distribution

Since metal layers are generally reflective in the wavelength range of interest in this work, we calculated the distribution of W/Al metal layers per structure across the full dataset. As shown in Figure S6, 49.49%, 35.42%, and 15.09% of all RMMs in the dataset consist of 0, 1, and $\geq$ 2 W/Al metal layers.

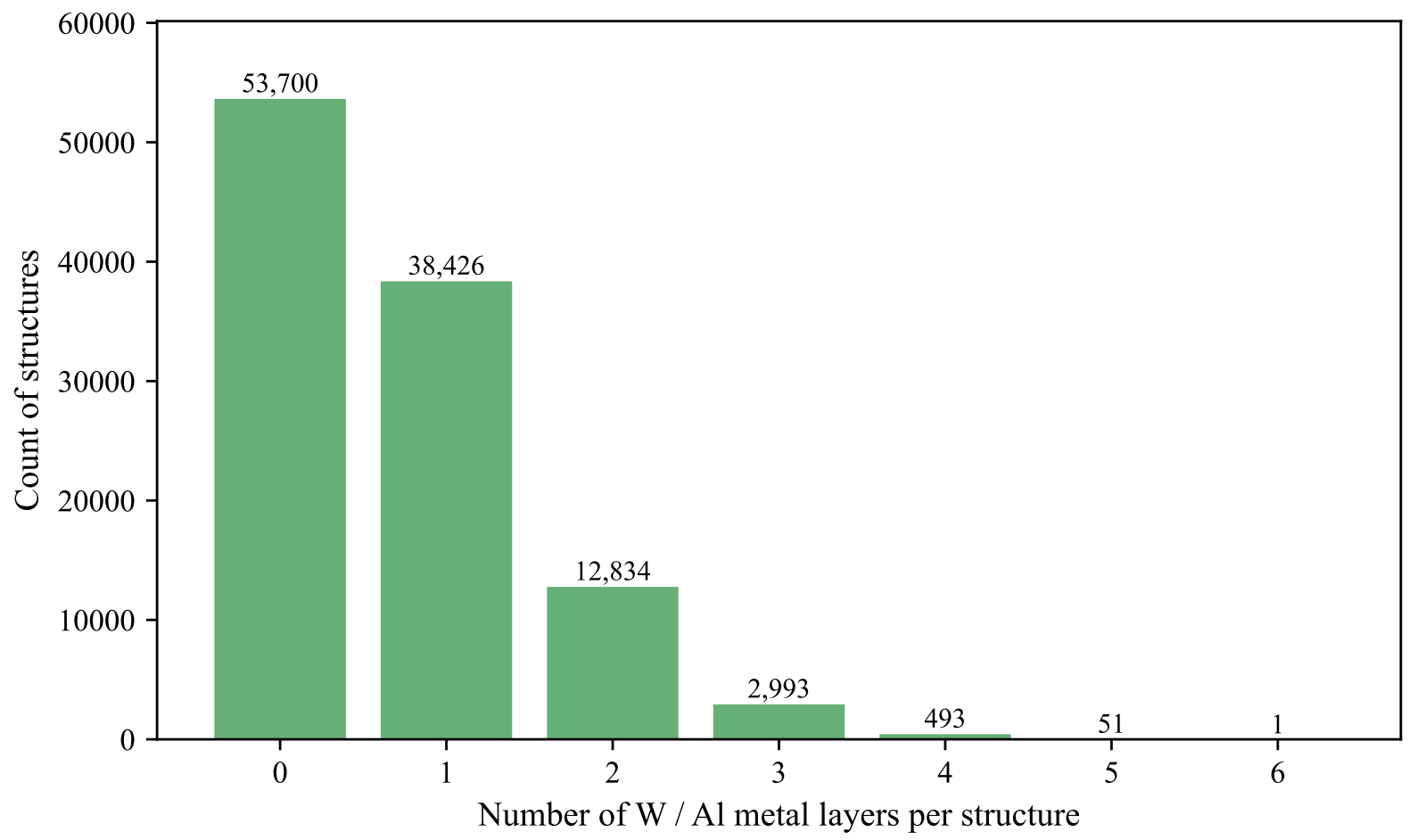

Figure S6: **Distribution of the number of W/Al metal layers per RMM structure.**

To characterize the transmissive behavior, we have also analyzed the pointwise transmissivity $T(\lambda)$ over all structures and sampled wavelengths, as shown in Figure S7.

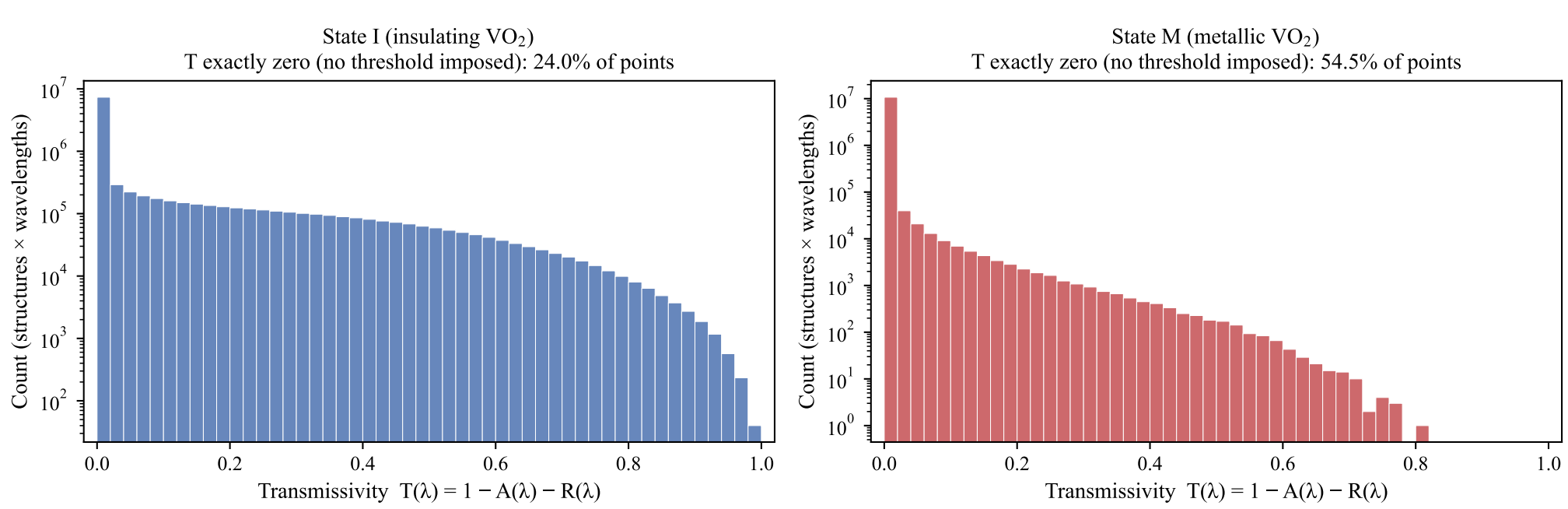

Figure S7: **State-resolved pointwise transmissivity distributions.**

Moreover, we evaluated the overall transmission for each RMM structure by calculating its band-maximum transmissivity($\max_\lambda T(\lambda)$). If this value is less than 0.1%, we define the corresponding RMM structure as opaque. We then counted the number of RMM structures that are considered as opaque accordingly. As shown in Figure S8, roughly 50.23% of the I-state RMM structures and 93.59% of M-state RMM structures are opaque.

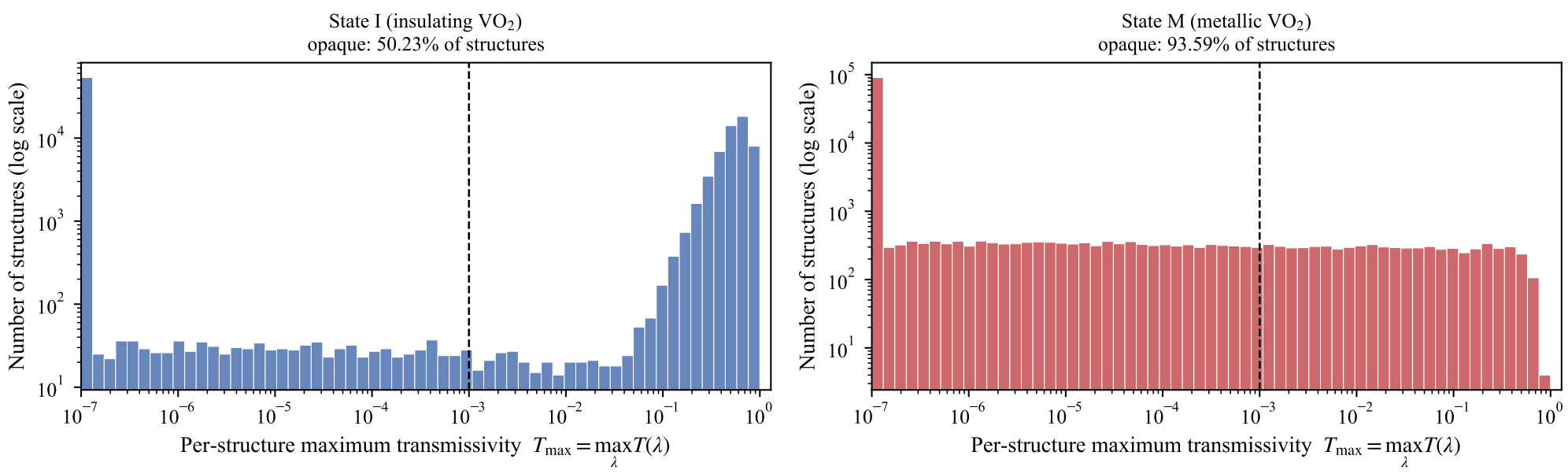

Figure S8: **State-resolved distributions of the per-structure maximum transmissivity.** The dashed line indicates the opacity threshold $T_{\max} = 10^{-3}$.

S4 Training and Inference

During the contrastive multi-state spectrum pretraining, a six-layer Transformer encoder [25] is trained as the spectrum encoder, with each layer's hidden dimension set to 768, aligning with the hidden size of GPT-2 [26]. The training is conducted with a batch size of 1,024 on a single NVIDIA GeForce RTX 3090 GPU for 1,000 epochs, with the initial temperature τ set to 0.07. Following the completion of pre-training, fine-tuning of GPT-2 is performed using eight NVIDIA GeForce RTX 3090 GPUs, with a batch size of 4 per GPU, for a total of 6,000 steps.
In inference, a greedy sampling strategy [27] is employed, selecting the token with the highest probability at each step to minimize randomness. Additionally, a fixed random seed is set for all subsequent experiments. As a result, all the experimental outcomes presented below are reproducible.
For optimization, in the Contrastive Multi-State Pretraining stage, training is performed using the Adam optimizer [28] with a learning rate of 3×10^{-4}. The fine-tuning of GPT-2 within CoSP is conducted utilizing the Hugging Face transformers library [29]. During the fine-tuning process of GPT-2, the AdamW optimizer [30] is employed with hyperparameters $\epsilon = 10^{-6}$, $\beta_1 = 0.9$, $\beta_2 = 0.999$, and a learning rate that begins at 2×10^{-4}, which decreases to zero following a cosine schedule.

S5 Baseline Modifications

None of the baselines compared in Table 2 of the main text is originally designed to handle the multi-state RMM inverse design problem, in which the objective is to simultaneously match a spectral pair corresponding to the two states (I and M) of a single underlying structure. To ensure a fair and technically meaningful comparison, method-specific adaptations are developed for every baseline, guided by the governing physics and the multi-state objective. Two shared adaptations are applied to all baselines: (1) the optimization target, reward, or loss is reformulated so that errors from both State I and State M spectra are aggregated into a single scalar (i.e., the sum of per-state MSE), thereby forcing the model to jointly fit both states of a common structure; (2) the structural parameterization is constrained to remain physically valid—layer thicknesses are bounded within the same fabrication-feasible range used in the dataset (30 nm to 5,000 nm), the layer count is kept within 5–12, and VO_2 is required to be present so that the two states are well defined. All learning-based baselines are trained from scratch on the same dataset used for CoSP. All neural-network baselines are trained on eight NVIDIA GeForce RTX 3090 GPUs.
Genetic Algorithm (GA) and Bayesian Optimization (BO). For both classical optimizers, each candidate RMM is encoded as a variable-length sequence of (material, thickness) pairs, with the layer count, materials, and thicknesses treated as mixed discrete–continuous decision variables within the ranges described above. The fitness/objective is the aggregated two-state spectral error evaluated via TMM. For GA, tournament selection, single-point crossover over the (material, thickness) sequence, and per-gene mutation that resamples either the material label or the thickness within its admissible range are adopted. For BO, a Gaussian-process surrogate over the same parameterization is built, with

expected-improvement (EI) used as the acquisition function. In the BO baseline, the MSE value shows no meaningful improvement across a window of consecutive trials at the end of optimization, while GA stops when it goes consecutive generations without a substantial gain in best fitness.

Deep Q-Learning Networks (DQN) and Proximal Policy Optimization (PPO). Both reinforcement-learning baselines construct the RMM in a sequential, layer-by-layer manner, where each action selects the material and thickness of the next layer under the same structural constraints as CoSP. The reward is the negative aggregated two-state spectral error, evaluated by TMM once the layered structure is complete. The state encodes the partial structure built up so far together with the two-state target spectrum, so that the policy is conditioned on both the design objective and the design history.

MLP / CNN / ResNet / Transformer Encoder (Discriminative Baselines). Each discriminative model takes the concatenated two-state target spectrum as input and directly regresses a structural vector encoding the materials and thicknesses of all layers. The training loss is the MSE between the predicted and ground-truth structural vectors in the dataset. At inference, the predicted structural vector is decoded into a valid layered RMM and evaluated by TMM. The four backbones (MLP, CNN, ResNet, Transformer encoder) are matched to a comparable parameter budget so that the comparison reflects architectural inductive biases rather than capacity.

OptoGPT. Since OptoGPT [31] is originally trained on a different dataset and targets single-state thin-film inverse design, the released checkpoint is not used directly. Instead, OptoGPT is re-trained from scratch on the multi-state dataset, feeding it the concatenated two-state target spectrum as input so that it produces a single RMM structure satisfying both target states, under the same training protocol as the other learning-based baselines.

Conditional GAN (cGAN). Following the formulation of Mirza and Osindero [32], the cGAN baseline is conditioned on the concatenated two-state target spectrum. The generator takes the spectrum together with a sampled noise vector as input and outputs the structural vector under the same fabrication-feasible parameterization as CoSP, while the discriminator takes the (spectrum, structure) pair and predicts whether the structure is real or generated. To stabilize training and avoid mode collapse, the adversarial loss is augmented with an L_2 structure-reconstruction term so that the generator is also driven to match the ground-truth structure rather than only to fool the discriminator. At inference, multiple noise vectors are sampled for each target spectrum, and the candidate whose TMM-evaluated two-state spectra best match the target is reported.

Conditional VAE (cVAE). Following Sohn et al. [33], the cVAE baseline encodes (spectrum, structure) pairs into a Gaussian latent space and decodes a structural vector from a latent code together with the same target spectrum. Both the encoder and the decoder are conditioned on the two-state spectrum, so that the latent space captures the structural diversity **given** a target spectrum (i.e., the one-to-many nature of inverse design) rather than absorbing the variation caused by different spectra. The training loss combines structure reconstruction with the standard KL term against an isotropic Gaussian prior. At inference, the encoder is discarded and multiple latent codes are drawn from the prior; the candidate whose TMM-evaluated two-state spectra best match the target is reported.

Together, these adaptations ensure that every baseline reported in Table 2 of the main text is evaluated as a solid, best-effort multi-state inverse designer.

S6 Extended Results

In this section, additional visualizations of inverse design samples are provided to further illustrate the performance of CoSP. **Figure S9** provides these additional examples of inverse design generated by CoSP. These figures showcase a variety of cases, highlighting the algorithm's accuracy and robustness across different scenarios.

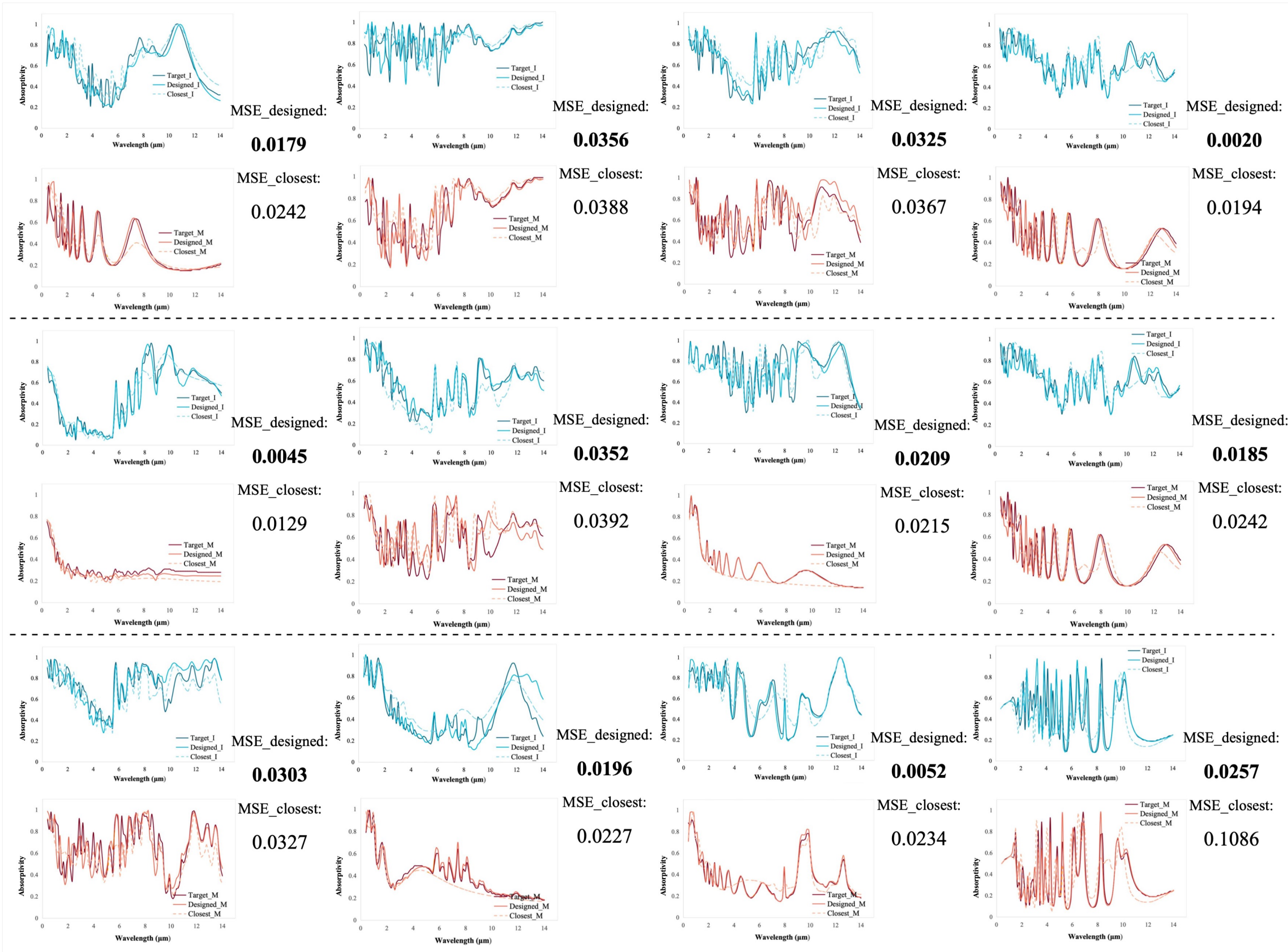

Figure S9: **Examples of inverse design results using CoSP.**

Moreover, to compare train-from-scratch and pretrained CoSP frameworks, we have conducted a controlled initialization study: the adopted train-from-scratch decoder is compared with the same decoder initialized using OpenAI-pretrained GPT-2 weights. As shown in Table S1, pretrained initialization provide worse performance in five out of six merics. Hence, the train-from-scratch configuration was adopted in this work.

Table S1: **Effect of GPT-2 decoder initialization under the same evaluation protocol.**

**Method**	**MSE ↓**	**MAE ↓**	**RMSE ↓**	R^2 ↑	**Corr. ↑**	**SID ↓**
Train from scratch	**0.0342**	**0.1181**	**0.1585**	**0.4290**	**0.7216**	0.3686
Pretrained weights	0.0479	0.1453	0.2012	0.3126	0.6563	**0.3390**

Additionally, we have evaluated the effect of contrastive multi-state spectrum pretraining by contrastive-pretraining ablation experiment on the full test set. As shown in Table S2, CoSP with contrastive pretraining consistently outperforms the corresponding variant without contrastive pretraining across all six metrics.

Table S2: **Contrastive-pretraining ablation on the complete test set.**

Method	MSE ↓	MAE ↓	RMSE ↓	R^2 ↑	Corr. ↑	SID ↓
CoSP w/ CP	**0.0342**	**0.1181**	**0.1585**	**0.4290**	**0.7216**	**0.3686**
CoSP w/o CP	0.0972	0.2221	0.2973	−0.3583	0.3928	0.5157

S7 Combining CoSP with Structural Optimization

Beyond directly producing usable RMM designs, CoSP can be flexibly combined with conventional structural optimizers as an optional refinement stage when a user wishes to further fine-tune a specific output. To illustrate this compatibility, a representative pipeline is investigated in which CoSP first generates an RMM, and Bayesian optimization is then applied on top of the CoSP output to adjust the layer thicknesses.

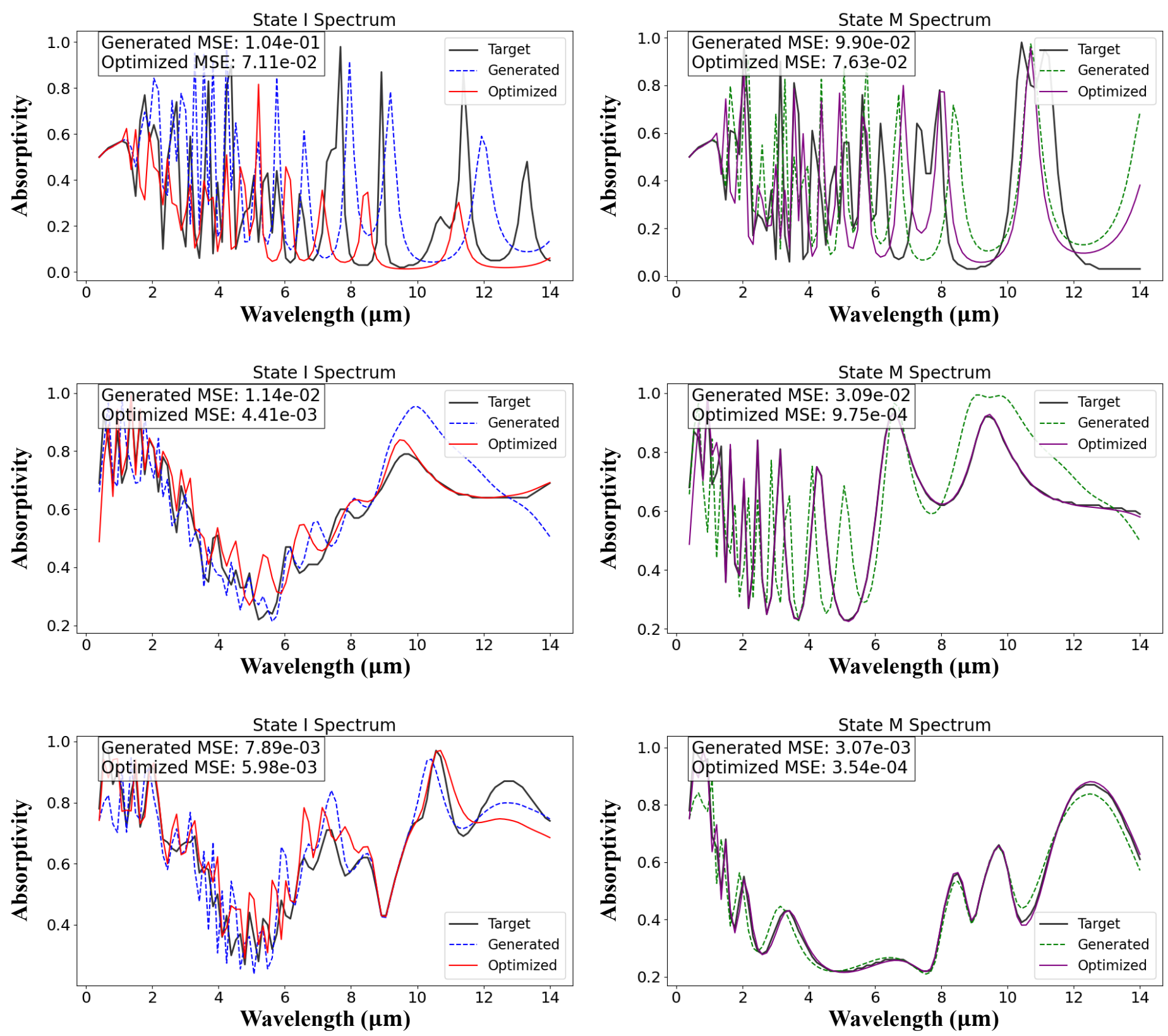

Figure S10: **Spectra of CoSP-designed devices before and after the optional optimization stage.**

Specifically, 500 target spectra are randomly drawn from the test set, and Bayesian optimization is applied to the corresponding CoSP outputs for structural-parameter refinement. As shown in **Figure S10**, this optional refinement step can further improve the spectral match for some samples. The associated cost is a substantially longer running time: CoSP itself produces a complete RMM design in 2.925 s on average, whereas 50 iterations of Bayesian optimization require 23.7 s—nearly an order of magnitude longer—and longer optimization budgets would amplify this gap. Moreover, the additional optimization stage does not uniformly outperform the original CoSP output across all samples. Therefore, CoSP itself is sufficient as a standalone inverse-design tool, while users with stricter accuracy requirements on a particular target can opt in to this refinement step at the cost of additional computation time.

References

[1] R. Unni, K. Yao, Y. Zheng, *ACS Photonics* **2020**, *7*, 10 2703.

[2] W. Xi, Y.-J. Lee, S. Yu, Z. Chen, J. Shiomi, S.-K. Kim, R. Hu, *Nature Communications* **2023**, *14*, 1 4694.

[3] W. Chen, Y. Gao, Y. Li, Y. Yan, J.-Y. Ou, W. Ma, J. Zhu, *Advanced Science* **2023**, *10*, 13 2206718.

[4] S. Yu, P. Zhou, W. Xi, Z. Chen, Y. Deng, X. Luo, W. Li, J. Shiomi, R. Hu, *Light: Science & Applications* **2023**, *12*, 1 291.

[5] J.-H. Song, P. Lalanne, M.-K. Seo, M. L. Brongersma, *ACS Photonics* **2023**, *10*, 8 2948.

[6] S. Byrnes, tmm 0.2.0: Simulate light propagation in multilayer thin and/or thick films using the fresnel equations and transfer matrix method, Python Package Index (PyPI), **2024**, URL `https://pypi.org/project/tmm/`.

[7] A. Yang, B. Yang, B. Hui, B. Zheng, B. Yu, C. Zhou, C. Li, C. Li, D. Liu, F. Huang, G. Dong, H. Wei, H. Lin, J. Tang, J. Wang, J. Yang, J. Tu, J. Zhang, J. Ma, J. Xu, J. Zhou, J. Bai, J. He, J. Lin, K. Dang, K. Lu, K. Chen, K. Yang, M. Li, M. Xue, N. Ni, P. Zhang, P. Wang, R. Peng, R. Men, R. Gao, R. Lin, S. Wang, S. Bai, S. Tan, T. Zhu, T. Li, T. Liu, W. Ge, X. Deng, X. Zhou, X. Ren, X. Zhang, X. Wei, X. Ren, Y. Fan, Y. Yao, Y. Zhang, Y. Wan, Y. Chu, Y. Liu, Z. Cui, Z. Zhang, Z. Fan, *arXiv Preprint arXiv:2407.10671* **2024**.

[8] T. Amotchkina, M. Trubetskov, D. Hahner, V. Pervak, *Applied Optics* **2020**, *59*, 5 A40.

[9] D. Fu, K. Liu, T. Tao, K. Lo, C. Cheng, B. Liu, R. Zhang, H. A. Bechtel, J. Wu, *Journal of Applied Physics* **2013**, *113*, 4 043707.

[10] M. R. Querry, Optical constants of minerals and other materials from the millimeter to the ultraviolet, Technical Report CRDEC-CR-88009, Chemical Research, Development and Engineering Center, U.S. Army, Aberdeen Proving Ground, Maryland, **1987**.

[11] M. Debenham, *Applied Optics* **1984**, *23*, 14 2238.

[12] C. A. Klein, *Applied Optics* **1986**, *25*, 12 1873.

[13] R. Boidin, T. Halenkovič, V. Nazabal, L. Beneš, P. Němec, *Ceramics International* **2016**, *42*, 1 1177.

[14] D. R. Smith, E. V. Loewenstein, *Applied Optics* **1975**, *14*, 6 1335.

[15] M. David, D. Disnan, A. Lardschneider, D. Wacht, H. T. Hoang, G. Ramer, H. Detz, B. Lendl, U. Schmid, G. Strasser, B. Hinkov, *Optical Materials Express* **2022**, *12*, 6 2168.

[16] X. Zhang, J. Qiu, X. Li, J. Zhao, L. Liu, *Applied Optics* **2020**, *59*, 8 2337.

[17] X. Zhang, J. Qiu, J. Zhao, X. Li, L. Liu, *Journal of Quantitative Spectroscopy and Radiative Transfer* **2020**, *252* 107063.

[18] J. Kischkat, S. Peters, B. Gruska, M. Semtsiv, M. Chashnikova, M. Klinkmüller, O. Fedosenko, S. Machulik, A. Aleksandrova, G. Monastyrskyi, Y. Flores, W. T. Masselink, *Applied Optics* **2012**, *51*, 28 6789.

[19] L. Gao, F. Lemarchand, M. Lequime, *Optics Express* **2012**, *20*, 14 15734.

[20] L. Y. Beliaev, E. Shkondin, A. V. Lavrinenko, O. Takayama, *Thin Solid Films* **2022**, *763* 139568.

[21] T. Siefke, S. Kroker, K. Pfeiffer, O. Puffky, K. Dietrich, D. Franta, I. Ohlídal, A. Szeghalmi, E.-B. Kley, A. Tünnermann, *Advanced Optical Materials* **2016**, *4*, 11 1780.

[22] A. D. Rakić, *Applied Optics* **1995**, *34*, 22 4755.

[23] W. S. M. Werner, K. Glantschnig, C. Ambrosch-Draxl, *Journal of Physical and Chemical Reference Data* **2009**, *38*, 4 1013.

[24] M. A. Ordal, R. J. Bell, R. W. Alexander, L. A. Newquist, M. R. Querry, *Applied Optics* **1988**, *27*, 6 1203.

[25] A. Vaswani, *Advances In Neural Information Processing Systems* **2017**.

[26] A. Radford, J. Wu, R. Child, D. Luan, D. Amodei, I. Sutskever, Language models are unsupervised multitask learners, Technical report, OpenAI, **2019**.

[27] E. Cohen, C. Beck, In *International Conference On Machine Learning*. PMLR, **2019** 1290–1299.

[28] D. P. Kingma, *arXiv Preprint arXiv:1412.6980* **2014**.

[29] T. Wolf, L. Debut, V. Sanh, J. Chaumond, C. Delangue, A. Moi, P. Cistac, T. Rault, R. Louf, M. Funtowicz, J. Davison, S. Shleifer, P. von Platen, C. Ma, Y. Jernite, J. Plu, C. Xu, T. L. Scao, S. Gugger, M. Drame, Q. Lhoest, A. M. Rush, In *Proceedings Of The 2020 Conference On Empirical Methods In Natural Language Processing: System Demonstrations*. Association for Computational Linguistics, **2020** 38–45.

[30] I. Loshchilov, F. Hutter, In *International Conference On Learning Representations*. **2017** .

[31] T. Ma, H. Wang, L. J. Guo, *Opto-Electronic Advances* **2024**, *7*, 7 240062.

[32] M. Mirza, S. Osindero, *arXiv Preprint arXiv:1411.1784* **2014**.

[33] K. Sohn, H. Lee, X. Yan, *Advances In Neural Information Processing Systems* **2015**, *28*.
